# Effect of cereal fermentation and carbohydrase supplementation on growth, nutrient digestibility and intestinal microbiota in liquid-fed grow-finishing pigs

**DOI:** 10.1038/s41598-020-70443-x

**Published:** 2020-08-13

**Authors:** Alberto Torres-Pitarch, Gillian E. Gardiner, Paul Cormican, Mary Rea, Fiona Crispie, John V. O’Doherty, Pierre Cozannet, Tomas Ryan, James Cullen, Peadar G. Lawlor

**Affiliations:** 1 Teagasc, Pig Development Department, Animal and Grassland Research and Innovation Centre, Moorepark, Fermoy, County Cork Ireland; 2 School of Agriculture and Food Science, University College Dublin, Belfield, Dublin Ireland; 3 Department of Science, Waterford Institute of Technology, Waterford, Ireland; 4 Animal and Bioscience Research Department, Animal and Grassland Research and Innovation Centre, Teagasc, Grange, County Meath Ireland; 5 Teagasc Food Research Centre, Moorepark, Fermoy, County Cork Ireland; 6 APC Microbiome Ireland, University College Cork, Cork, Ireland; 7 Adisseo France SAS, Antony, France

**Keywords:** Applied microbiology, Microbiome, Animal physiology

## Abstract

This study aimed to determine the impact of fermenting the cereal fraction of the diet (C_ferm_) and enzyme supplementation (ENZ) on the bacterial composition of the feed, nutrient digestibility, pig growth, feed efficiency (FE), intestinal volatile fatty acid (VFA) concentrations and intestinal microbiota composition. A total of 252 grow-finisher pigs (~ 40.4 kg; 7 pigs/pen) were randomly allocated to 4 diets in a 2 × 2 factorial arrangement for 55d. The diets were: (1) fresh liquid feed (Fresh); (2) C_ferm_ liquid feed (Ferm); (3) Fresh + ENZ and (4) Ferm + ENZ. C_ferm_ increased total tract nutrient digestibility, reduced caecal butyrate and propionate concentrations, and increased average daily gain (ADG). ENZ increased ileal and total tract nutrient digestibility, reduced caecal isobutyrate and propionate concentrations, and improved FE. Bacterial taxa positively correlated with pig growth (*Lactobacillus kisonensis* in the ileum and *Roseburia faecis* in the caecum) were more abundant in pigs fed ENZ diets, whereas most of the ileal bacterial taxa negatively correlated with growth (*Megasphaera, Bifidobacterium and Streptococcus*) had lower abundance in pigs fed C_ferm_ diets. In conclusion, C_ferm_ increased ADG and ENZ improved FE, with these improvements possibly mediated by increased nutrient digestibility, and beneficial modulation of the intestinal microbiota.

## Introduction

Improving feed efficiency (FE) reduces the environmental impact and increases the profitability of pig production. Despite the improvements in pig FE achieved in recent years^1,2^, it still takes ~ 3.5 kg of feed to produce 1 kg of saleable pig meat^2–4^. Nutritional strategies can be implemented to improve FE; however, the impact of these strategies on the microbial composition of the gastrointestinal tract (GIT) is seldom explored. This is surprising given the influence of the intestinal microbiota on the digestion and absorption process and as a consequence FE in pigs^5–10^.

In the current study, two nutritional strategies to improve FE were studied: firstly, fermentation of the cereal fraction of the diet (C_ferm_) and secondly, dietary inclusion of an exogenous carbohydrase enzyme complex containing xylanase and β-glucanase (ENZ). Cereal fermentation has been suggested as a strategy to increase dietary nutrient digestibility and favourably modulate the intestinal microbiota of pigs, while minimizing the degradation of dietary amino acids (AA) and the resultant production of undesirable substances such as biogenic amines^11,12^ which can be a feature of whole diet fermentation. Cereal fermentation has been demonstrated to increase lactic acid bacteria (LAB) counts and consequently the concentration of lactic acid, both of which help to control the growth of potentially pathogenic bacteria, both in the feed and the GIT of pigs^11,13–16^. However, its impact on the full bacterial profile of either has yet to be reported. Studies have shown that addition of ENZ to pig diets can improve nutrient digestibility, increase growth and improve FE^17–25^. The ENZ complex can break down arabinoxylans and β-glucans, present in the fibrous fraction of the diet, into substrates (monomers of xylose, arabinose and glucans) that can provide energy for the pig directly via absorption in the small intestine or indirectly via microbial fermentation into volatile fatty acids (VFA) in the large intestine which are then absorbed^17^. A large number of publications have examined the effects of dietary ENZ supplementation on the FE of grow-finisher pigs^18–25^; however, improvements are not consistently observed^26,27^. Dietary ENZ supplementation can influence the abundance of selected enteric bacterial groups (e.g. *Bifidobacterium* spp. and *Lactobacillus* spp.) in pigs^24,28–30^. However, the microbial species that most efficiently utilise xylose, arabinose and glucans are unknown. The variability in FE response to ENZ supplementation may be explained by unveiling the effects of ENZ dietary supplementation on intestinal bacterial taxa associated with pig growth traits.

Liquid feeding involves mixing and distributing feed to pigs in liquid form. In its simplest form, dry feed is mixed with water, at a pre-determined water to feed ratio prior to feeding. As well as many other possibilities, the liquid feed can be fed fresh (Fresh) or the cereal component of the diet can be pre-fermented before mixing and feeding the liquid diet (C_ferm_). The types of carbohydrates available as microbial substrates in liquid feed and within the pig’s intestinal digesta are likely to change due to C_ferm_ and ENZ supplementation. The hypothesis of this study was that feeding C_ferm_ diets, with or without ENZ supplementation, to grow-finishing pigs would favourably modulate the intestinal microbiota and consequently improve growth and FE. The objective was to determine the impact of both strategies on the bacterial composition of the feed, nutrient digestibility, pig growth, FE, intestinal VFA concentrations and intestinal microbiota composition. To help identify the microbial taxa that utilize the substrates released by C_ferm_ and ENZ most efficiently, correlations between growth parameters, intestinal VFA concentrations and the relative abundance of microbial taxa were investigated.

## Material and methods

Experimental facilities, experimental set-up, analysis of feed, faeces and digesta samples, and statistical analyses were essentially as described by Torres-Pitarch et al.^31^ except that in the current experiment the cereal fraction of the diet was fermented for an initial 52 h period and thereafter back-slopped (fermentation technique in which some of the previous fermentate is used as the inoculum for the next cereal fermentation) daily throughout the feeding trial. An initial fermentation time of 52 h was chosen based on best practice used in the pig industry.

### Experimental design

A total of 252 pigs [Maxgrow × (Landrace × Large White); Hermitage Genetics, Sion Road, Kilkenny, Ireland] with an initial live weight (LW) of 40.6 (± 0.50 SEM) kg were penned in groups of 7 pigs of the same sex per pen. There were a total of 9 replicates (5 male and 4 female) for each dietary treatment and each replicate comprised a single sex group of 7 pigs/pen. The duration of the experiment was 55d and the experiment was arranged as a 2 × 2 factorial. The factors were: fermentation of the cereal fraction (C_ferm_; un-fermented vs. fermented) and supplementation of cereals with ENZ [− vs. +]. This resulted in four dietary treatments: (1) fresh liquid feed (Fresh); (2) C_ferm_ liquid feed (Ferm); (3) Fresh + ENZ and (4) Ferm + ENZ. All diets were formulated to contain 9.4 MJ net energy/kg (7% below the NRC recommendation^32^) and 9.15 g/kg of standardised ileal digestible (SID) lysine [at the NRC recommendation^32^ (Table 1)]. All other AA were formulated relative to lysine according to the ideal protein concept^32^. The ENZ (Rovabio Excel AP, Adisseo France SAS, Antony, France) was derived from *Talaromyces versatilis* sp. and provided 22,000 viscosity units (VU) of endo-1,4-β-xylanase (EC 3.2.1.8) and 30,000 VU of endo-1,3(4)-β-glucanase (EC 3.2.1.6) per gram of powder product. The ENZ was supplemented to the cereal fraction of the diet at 120 g/tonne of cereal mix in order to provide the manufacturer’s recommended inclusion level of 100 g/tonne of finished feed (2,200 VU of Xylanase and 3,000 VU of β-glucanase per kg of finished diet, 88% dry matter [DM] basis).

**Table 1:** Ingredient and nutrient composition of dietary components and experimental diets^a^ (on an air dry basis, g/kg unless otherwise stated).

	Dietary components			Experimental diets	
	CER	CER + ENZ	BAL	Basal	Basal + ENZ
**Ingredient composition**^**b**^					
Barley	450.60	450.48	0.00	377.30	377.19
Wheat	418.00	418.00	0.00	350.00	349.99
Soya bean meal	0.00	0.00	829.60	135.00	134.97
Wheat pollard	131.40	131.40	0.00	110.00	110.02
Limestone	0.00	0.00	76.80	12.50	12.50
Lysine	0.00	0.00	26.90	4.40	4.37
Mono dicalcium phosphate	0.00	0.00	22.60	3.70	3.68
Salt	0.00	0.00	18.40	3.00	3.00
L-threonine	0.00	0.00	6.70	1.10	1.09
Soya oil	0.00	0.00	6.10	1.00	1.00
Vitamin and mineral premix^c^	0.00	0.00	6.10	1.00	1.00
DL-methionine	0.00	0.00	4.90	0.80	0.80
Celite	0.00	0.00	1.80	0.30	0.30
Enzyme^d^	0.00	0.12	0.00	0.00	0.10
**Component composition**^**b**^					
Cereal fraction	1,000.00	1,000.00	0.00	837.30	837.30
Balancer fraction	0.00	0.00	1,000.00	162.70	162.70
**Nutrient composition**^**e**^					
Dry matter	866.00	867.00	899.00	871.00	872.00
Crude protein	103.00	106.00	423.00	155.00	157.00
Ash	21.00	22.00	174.00	45.900	46.70
Oil	27.00	27.00	28.00	27.20	27.20
Crude fibre	39.00	39.00	28.00	37.20	37.20
Neutral detergent fibre	139.00	134.00	67.00	127.30	123.10
Acid detergent fibre	53.00	51.00	43.00	51.60	49.70
Net energy, MJ/kg	–	–	–	9.40	9.40
Total lysine	3.60	3.60	40.20	9.55	9.55
SID^f^ Lysine	–	–	–	9.15	9.15
Total Ca	–	–	–	6.48	6.48
Digestible P	–	–	–	2.40	2.40
Xylanase activity^g^, VU/kg	0.00	5,712	–	0.00	4,783
β-glucanase activity^g^, VU/kg	0.00	5,542	–	0.00	4,640

^*a*^*CER* cereal fraction of the diet, *CER + ENZ* cereal fraction of the diet supplemented with a carbohydrase enzyme (xylanase and β-glucanase, ENZ), *BAL* balancer fraction (non-cereal component) of the diet.

^b^Calculated values.

^c^Vitamin and mineral premix provided per kilogram of complete diet (on an air dry basis): Cu from copper sulphate, 15 mg; Fe from ferrous sulphate monohydrate, 24 mg; Mn from manganese oxide, 31 mg; Zn from zinc oxide, 80 mg; I from potassium iodate, 0.3 mg; Se from sodium selenite, 0.2 mg; retinyl acetate 0.7 mg; cholecalciferol, 12.5 μg; DL-alpha-tocopheryl acetate, 40 mg; Vitamin K, 4 mg; vitamin B12, 15 μg; riboflavin, 2 mg; nicotinic acid, 12 mg; pantothenic acid, 10 mg; vitamin B1, 2 mg; vitamin B6, 3 mg; an0d celite, 300 mg.

^d^Carbohydrase complex based on xylanase and β-glucanase (Rovabio Excel AP, Adisseo France SAS, Antony, France) providing a minimum guaranteed content of 2,200 VU and 3,000 VU, respectively, per kg of finished diet (on an air dry basis).

^e^Analysed values for dietary components (CER, CER + ENZ and BAL). For the experimental diets (basal and basal + ENZ) values given are calculated from the analysed dietary component values. Values with a “–” were not analysed and the calculated values given for the experimental diets are from the calculated values in the matrix formulation.

^f^*SID* = Standardized ileal digestibility.

^g^One viscosity unit (VU) is defined as the amount of enzyme reducing the viscosity of the solution, to give a change in relative fluidity of 1 dimensionless unit per minute per g at pH 5.5 and 30 °C.

### Feed preparation and animal management

Three dietary components were manufactured in meal form at the Teagasc feed mill (Teagasc, Moorepark, Fermoy, Co. Cork, Ireland): (1) Cereal fraction (CER) composed of a mix of barley (45%), wheat (42%) and wheat feed (12%) which were ground through a 3 mm screen before mixing; (2) CER supplemented with the ENZ complex (CER + ENZ) and (3) Balancer fraction (BAL) consisting of a mixture of soya bean meal, synthetic AA, vitamins and minerals. The three dietary components were transported to the adjoining experimental farm and stored in steel bins during the experimental period. The liquid-fed dietary treatments were prepared and provided to the pigs at the experimental farm. The ingredient and nutrient composition of the dietary components and the basal diets are reported in Table 1. The computerised liquid feeding system (HydroMix, Big Dutchman, Germany) consisted of two fermentation tanks (2000 L), 2 mixing tanks (500 L), each equipped with an agitator (consisting of 1 vertical axis and 6 horizontal blades) and a high-pressure air system equipped for delivery of the feed from the mixing tanks to the pen troughs, each of which was fitted with a solenoid valve and an electronic feed sensor. Feed level in the troughs was checked by sensor 6 times per day and feed was prepared and delivered to the troughs which had feed below the level of the sensor at these times. To prepare the fresh liquid dietary treatments (Fresh and Fresh + ENZ), the CER (or CER + ENZ) and BAL at the correct ratio (0.837:0.163, CER:BAL) were mixed with water at a ratio of 1:2.5 (fresh feed:water, 25.1% DM) and agitated for 5 min before delivery to the troughs. To prepare the C_ferm_ dietary treatments (Ferm and Ferm + ENZ), the CER or CER + ENZ was mixed with water (25.1% DM) and fermented with agitation for an initial set-up period of 52 h at the start of the experiment. The Ferm and the Ferm + ENZ were mixed with the BAL and agitated for 5 min before delivery of liquid feed to the troughs. Thereafter, during the experiment, the quantity of feed consumed by the pigs was replenished daily to keep a constant level of 2000 L in the fermentation tanks. The CER and CER + ENZ components in the fermentation tanks were agitated for cycles of 30 min agitation and 15 min stationary. At time zero of fermentation an inoculant containing *Lactobacillus plantarum* DSMZ16627 and *Pediococcus acidilactici* NCIMB3005 (Sweetsile; Agway, Aherla, Co. Cork, Ireland) was added to the fermentation tanks at a dose sufficient to obtain an initial count of ~ 5 × 10^5^ CFU of each strain per mL of liquid cereal. This is a silage inoculant and was chosen as no commercially available liquid feed inoculant was available at the time. Also, *L. plantarum* and *P. acidilactici* are dominant LAB species commonly found in fermented liquid feed^11,12,15^. The fermentation tanks and mixing tanks were located in a room where the temperature was maintained at 20 to 23 °C.

The groups of 7 pigs were penned in slatted pens (2.37 m × 2.36 m) with solid PVC partitions. The feeders were short stainless steel troughs (100 cm × 32.5 cm × 21 cm) located on top of a rubber mat (1.5 × 1 m) to help minimise feed wastage. Each pen was provided with a drinking bowl (DRIK-O-MAT, Egebjerg International A/.S, Egebjerg, Denmark). Air temperature was maintained at 20 to 22 °C. Pigs were observed closely twice daily. Any pig showing signs of ill-health was treated as appropriate. All veterinary treatments were recorded including identity of pig, symptom, medication used and dosage.

### Recordings and sample collection

Individual pig LW and feed disappearance per pen was recorded on d0, d14, d28, and d55 of the experiment, from which ADG, ADFI and FCR were calculated. At time 0 h, 12 h, 24 h and 52 h of fermentation (during the initial experimental set-up period) and at d10 and d52 of the experiment, ~ 600 g of the fermented CER and the fermented CER + ENZ were collected from the fermentation tanks and pH and temperature were recorded (Figure S1 online). Approximately 600 g of each of the dry dietary components and the dietary treatments were collected from the storage silos, the mixing tank and the pen troughs at d10 and d52 of the experiment to determine how the microbial content of the mixing tanks and troughs in particular changed over time. Samples from the pen troughs were collected 30 min before a new feed mix was dispensed into the trough. One aliquot (~ 5 mL) of each feed sample was immediately snap-frozen in liquid nitrogen and stored at − 80 °C for subsequent microbiota analysis. A second aliquot of the sample (~ 20 mL) was stored at − 20 °C for subsequent VFA analysis. A third aliquot of the sample (~ 50 mL) was stored on ice for microbiological analysis on the day of collection (within 3 h). The other feed and faecal sampling procedures and storage of samples were performed as outlined by Torres-Pitarch et al.^31^. Briefly, after the feed trough sample aliquots above were taken, the rest of the sample was frozen at − 20 °C in aluminium foil trays for subsequent freeze-drying prior to chemical analysis. Fresh faecal samples from 6 pens (pooled from 3 pigs/pen) per treatment were collected daily for two days prior to slaughter and the corresponding feed for each pen was collected 1 day before faecal collection. Feed and faecal samples were stored at − 20 °C for subsequent apparent total tract digestibility (ATTD) determination. Slaughter of pigs and sampling at slaughter were also performed as described by Torres-Pitarch et al.^31^ as follows. At d55 of the experiment, pigs were transported ~ 100 km to a commercial abattoir (Dawn Pork and Bacon, Waterford, Ireland), stunned with CO_2_ and killed by exsanguination. At slaughter, the intestinal tracts of 22 pigs per treatment (2 and 3 pigs per pen of males and females, respectively) were recovered. Digesta samples were collected from the terminal ileum (1.5 m proximal to the ileo-caecal valve) and the terminal end of the caecum. Three aliquots of ileal digesta were stored as for feed samples: one aliquot (~ 5 mL) for microbiota analysis, a second aliquot (~ 20 mL) for VFA analysis and the remainder for apparent ileal digestibility (AiD) determination. Two aliquots of caecal digesta were stored; one for microbiota analysis (~ 5 mL; snap-frozen in liquid N and stored at − 80 °C) and a second for VFA analysis (~ 20 mL, − 20 °C storage). Carcass measurements were taken as described by Torres-Pitarch et al.^31^ as follows. Hot carcass weight was recorded 45 min after stunning, and back-fat thickness and muscle depth measured at 6 cm from the edge of the split back at the level of the 3rd and 4th last rib were determined using a Hennessy Grading Probe (Hennessy and Chong, Auckland, New Zealand). Lean meat content was estimated according to the following formula: Estimated lean meat content (%) = 60.3 – 0.847x + 0.147y where x = fat depth (mm); y = muscle depth (mm) (Department of Agriculture Food and Rural Development, 2001).

### Feed analysis

The dietary components (CER, CER + ENZ, and BAL) were ground through a 1 mm screen in a Cyclotec™ mill (FOSS electric, Hilleroed, Denmark) and analysed for DM, ash, fat, gross energy (GE), crude fibre (CF), neutral detergent fibre (NDF), acid detergent fibre (ADF) and crude protein (CP) as described by Clarke et al.^17^ and Torres-Pitarch et al.^33^. Amino acid concentrations were determined using high performance liquid chromatography^34^. The liquid feed samples collected from the fermentation tanks and the pen troughs were freeze-dried prior to grinding through a 1 mm screen and analysed as outlined above, as well as for biogenic amines. Biogenic amines were analysed by Sciantec Ltd. (Cawood, North Yorkshire, United Kingdom) by extraction with 10% trichloroacetic acid solution and subsequent ion exchange chromatography. The cereal dietary components (CER and CER + ENZ) were analysed by ADISSEO France for xylanase and β-glucanase activity using a colorimetric assay. One VU of endo-1,4-β-xylanase activity was defined as the amount of enzyme reducing the viscosity of the solution, to give a change in relative fluidity of 1 dimensionless unit per minute per mL (or per g) under the conditions of the assay (pH 5.5 and 30 °C). One VU of endo-1,3(4)-β-glucanase activity was defined as the amount of enzyme reducing the viscosity of the solution, to give a change in relative fluidity of 1 dimensionless unit per minute per mL (or per g) under the conditions of the assay (pH 5.5 and 30 °C).

### Nutrient digestibility analysis

The freeze-dried feed, faeces and ileum digesta samples collected for digestibility determination were individually ground through a 1 mm screen using the Cyclotec™ mill. After milling, each sample type was pooled by pen (n = 9 per treatment) and analysed for DM, ash, acid insoluble ash (AIA), GE and CP for determination of AiD and ATTD. The concentration of AIA was determined according to the method of McCarthy et al.^35^.

### Volatile fatty acid analysis and pH of feed and ileal and caecal digesta samples

Fermented cereals, liquid feed, and ileal and caecal digesta samples were thawed to room temperature and pH was measured using a pH meter (F2-Meter, Mettler Toledo, Germany). Volatile fatty acid concentrations were analysed in duplicate in these samples using gas liquid chromatography according to the method described by Clarke et al.^17^ but instead of ~ 1 g of initial sample ~ 3.5 g was used for the extraction.

### Microbiological analysis of liquid feed, ileum and caecum digesta samples

Feed samples collected from the fermentation tanks and at the pen troughs (10 g) were homogenized in 90 mL of maximum recovery diluent (MRD) and a tenfold dilution series was performed in MRD. Appropriate dilutions were plated in duplicate as follows; (1) pour-plated on De Man, Rogosa and Sharpe (MRS) agar containing 50 U/mL nystatin (Sigma-Aldrich, Arklow, Co. Wicklow, Ireland), overlaid and incubated at 30 °C for 72 h for LAB; (2) pour-plated on Violet Red Bile Dextrose (VRBD) agar, overlaid and incubated at 37 °C for 24 h for *Enterobacteriaceae*; and (3) spread-plated on Yeast Glucose Chloramphenicol (YGC) agar incubated at 25 °C for 5d for yeasts and moulds. Colonies were counted, and the counts averaged and presented as CFU/g of the original sample. All microbiological media were obtained from Merck (Darmstadt, Germany). Total DNA was extracted from the fermented cereals, liquid feed (from mixing tanks and pen troughs), ileal and caecal samples using the QIAamp DNA stool minikit (Qiagen, Crawley, United Kingdom) according to the manufacturer’s instructions, apart from adding a bead beating step after sample addition to the InhibitEX buffer and increasing the lysis temperature to 95 °C to increase the DNA yield^36^. Microbial profiling was performed using high-throughput sequencing of the V3-V4 region of the 16S rRNA gene (paired end reads of 250 bp) on an Illumina MiSeq platform according to the standard Illumina protocol, except that the PCR mix volume was doubled in the first PCR step and 30 cycles were used instead of 25^37^. Paired-end reads in all samples were quality assessed using FastQC v0.11.7. BBduk from the BBTools suite (https://jgi.doe.gov/data-and-tools/bbtools/) was used to quality trim (cuttoff-phred = 20). Primers and low quality read tails were also removed. The DADA2 pipeline was used to perform read filtering and de-replication, chimera detection and removal, read-pair merging and inference of amplicon sequence variants (ASV) in each sample. Taxonomy was assigned to each derived ASV using a naive Bayesian classifier method against the Silva database (Version 128). Species level was identified, when possible, by blasting the sequences against the nucleotide database of the U.S. National Center for Biotechnology Information (NCBI). Alpha diversity indices (Chao1, Shannon and Simpson) based on subsampled read data (n = 32,500 reads per sample) and β-diversity (Bray–Curtis) analyses were calculated using the phyloseq package in R^38^. Data were subsequently plotted using the ggplot2 package in R^38^.

### Statistical analysis

Growth parameters (LW, ADG, ADFI and FCR), carcass quality parameters, nutrient digestibility, digesta pH and VFA concentrations were analysed using the PROC MIXED procedure of SAS software version 9.4 (SAS Institute, Inc., Cary, NC, US). For growth parameters; C_ferm_, ENZ supplementation, time, sex and their associated interactions were included in the model as fixed effects while initial LW was included as a covariate in the model and day was regarded as a repeated variable with pen as the experimental unit. For carcass quality parameters, nutrient digestibility, digesta pH and VFA concentrations: C_ferm_, ENZ supplementation, sex and their associated interactions were included in the model as fixed effects with pen as the experimental unit; for kill out percentage, muscle depth, fat depth and lean meat percentage, carcass weight was included as a covariate in the model. A compound symmetry covariance structure was fitted to data describing growth parameters (LW, ADG, ADFI and FCR). Model suitability was investigated by checking normality of scaled residuals using the Shapiro–Wilk test within the UNIVARIATE procedure of SAS. The results were presented as least square means ± SEM. Microbial relative abundances at phylum, family, genus and ASV levels were analysed using generalized linear mixed model equation methods in the PROC GLIMMIX procedure of SAS. Each taxon was compared in a univariate manner and the p-values were corrected for multiple comparisons using a Benjamini–Hochberg estimated false discovery rate (FDR). A gamma distribution was assumed for all data. Models included C_ferm_, ENZ supplementation and their interaction as fixed effects. In all models, data were back transformed to the original distribution using the ilink option. Spearman correlations between the differentially abundant genera and ADG, carcass quality data, digesta pH and digesta VFA concentrations were performed using base R^38^ using the individual pig as the experimental unit. Significance was reported for P ≤ 0.05.

### Ethics approval

Ethical approval for this study was granted by the Teagasc Animal Ethics Committee (approval no. TAEC86/2015). The experiment was conducted in accordance with Irish legislation (SI no. 543/2012) and EU Directive 2010/63/EU on the protection of animals used for scientific purposes.

## Results

### Characterization of fermented cereals and dietary treatments sampled at different time points from the fermentation tanks, the mixing tank and the troughs

The calculated and analysed composition of the dry dietary components [balancer (BAL), cereals (CER) and cereals supplemented with enzyme (CER + ENZ)] and the basal diets are presented in Table 1. The analysed values for fat, CF, NDF, CP and lysine were as expected. The concentrations of AA and VFA, pH, and selected microbial counts in the fermented CER and CER + ENZ collected from the fermentation tanks at the different time points are presented in Table 2. The pH and temperature from the fermentation tanks are reported in supplementary Table S1 online.

**Table 2:** Analysed amino acid (AA) composition (n = 1, %), pH (n = 1), volatile fatty acid (VFA) concentrations (n = 1, mmol/kg) and microbial counts (n = 2, log_10_ CFU/g) of the cereal fraction (CER) and the cereal fraction supplemented with xylanase and β-glucanase (CER + ENZ) collected from the fermentation tanks during the initial fermentation (0–52 h) and during the feeding trial (d10, d51).

	CER						CER + ENZ						
	0 h	12 h	24 h	52 h	d10	d51	0 h	12 h	24 h	52 h	d10	d51	SD^a^
**AA composition**													
Lysine	0.49	0.41	0.39	0.37	0.37	0.39	0.40	0.39	0.36	0.38	0.48	0.35	–
Threonine	0.34	0.37	0.36	0.38	0.36	0.37	0.38	0.39	0.36	0.38	0.43	0.35	–
Methionine	0.15	0.19	0.18	0.19	0.19	0.19	0.18	0.19	0.18	0.18	0.22	0.17	–
Valine	0.46	0.55	0.53	0.55	0.53	0.52	0.51	0.51	0.50	0.51	0.60	0.49	–
Leucine	0.65	0.78	0.75	0.75	0.73	0.76	0.76	0.76	0.75	0.76	0.86	0.70	–
Isoleucine	0.38	0.41	0.39	0.41	0.40	0.40	0.39	0.38	0.39	0.39	0.47	0.36	–
pH	6.34	6.09	4.47	3.70	3.87	3.78	6.31	6.30	5.43	3.76	3.92	3.73	–
**VFA concentrations**													
Acetate	29.55	16.81	20.23	27.30	28.09	50.93	20.96	16.62	23.88	23.37	85.14	84.66	–
Propionate	0.64	0.45	0.30	0.35	0.30	0.81	0.54	0.34	0.36	0.20	0.42	1.53	–
Isobutyrate	1.43	1.36	1.17	1.01	0.75	1.16	1.41	1.61	1.56	1.05	1.02	1.34	–
Butyrate	0.51	0.40	0.23	0.25	0.15	0.30	0.47	0.31	0.25	0.16	0.31	0.28	–
Isovalerate	0.13	0.12	0.07	0.08	0.03	0.08	0.14	0.13	0.14	0.07	0.14	0.08	–
Valerate	0.30	0.19	0.08	0.10	0.05	0.14	0.25	0.17	0.15	0.06	0.14	0.17	–
Total VFA	32.55	19.03	22.08	29.09	29.37	53.40	23.77	19.18	26.35	24.90	87.18	88.05	–
Acetate:Propionate	45.88	37.50	67.32	78.41	94.19	63.28	38.59	48.44	66.63	115.63	202.20	55.50	–
Protein-derived VFA^b^	1.85	1.66	1.32	1.19	0.82	1.37	1.80	1.91	1.86	1.17	1.30	1.58	–
**Microbial count**^**c**^													
Lactic acid bacteria	5.1	8.5	9.1	9.2	9.1	9.2	5.7	6.2	8.7	8.8	9.0	9.0	0.18
Enterobacteriaceae	5.2	7.0	4.9	< DL	< DL	< DL	5.3	6.0	7.7	< DL	< DL	< DL	0.13
Yeasts	4.9	5.0	5.2	6.8	6.1	6.3	3.8	4.2	5.0	4.8	6.8	6.7	0.20
Moulds	< DL	< DL	< DL	< DL	< DL	< DL	< DL	< DL	< DL	< DL	< DL	< DL	–

^a^*SD* standard deviation.

^b^Protein-derived VFA calculated as the sum of isobutyrate, isovalerate and valerate.

^c^DL = Detection limit (2 log_10_ CFU/g for *Enterobacteriaceae* and 3 log_10_ CFU/g for yeasts and moulds).

The concentrations of AA did not change in the fermentation tanks, either during the initial 52 h fermentation or over time during the feeding trial. There were some differences between the tank containing the fermented CER and the tank containing the fermented CER + ENZ during the initial fermentation process, in that the LAB counts did not increase as fast and the pH and *Enterobacteriaceae* counts did not decrease as fast in the CER + ENZ tank. However, at time 52 h, similar results were observed in both fermentation tanks for pH, VFA concentrations and LAB*, Enterobacteriaceae*, and mould counts but yeast counts were 100-fold lower in the CER + ENZ tank (initial yeast counts were also lower in this tank). On average, in both fermentation tanks, from time 0 h to 52 h; the pH decreased from 6.3 to 3.7, LAB counts increased from 5.2 to 9.2 log_10_ CFU/g, *Enterobacteriaceae* counts decreased from 5.2 log_10_ CFU/g to below the detection limit (2.0 log_10_ CFU/g), yeast counts increased from 4.4 to 5.8 log_10_ CFU/g and the total concentration of VFA increased from 21.5 to 70.8 mmol/kg. Similar values to those found at 52 h of fermentation were found for pH, LAB, *Enterobacteriaceae*, and moulds at d10 and d51 of the study. However, yeast counts increased in the CER + ENZ tank during the feeding trial and total VFA concentrations (mainly accounted for by acetate) increased in both tanks, although the increase was seen earlier in the CER + ENZ tank. In addition, the total concentration of VFA was higher in this fermentation tank at both d10 and d51 (Table 2). The analysed chemical composition, pH, biogenic amine and VFA concentrations and microbial counts of the diets collected from the pen troughs are presented in Table 3. The analysed values for fat, GE, CF, CP and lysine were as expected. *Enterobacteriaceae* were detected in the pen troughs, whereas they were undetectable in the fermentation tanks. However, the analysed chemical composition, pH and selected microbial counts of the diets were similar between dietary treatments (Table 3). The concentration of total VFA was higher, mainly due to increased acetate concentration, in troughs containing feed supplemented with ENZ than in troughs containing un-supplemented feed (54.7 vs. 36.5 mmol/g, SD = 5.44, n = 2), similar to what was found for the fermentation tanks. Biogenic amine concentrations in the pen troughs were low and/or below the detection limit in most cases (Table 3). The highest cadaverine and putrescine concentrations were found in pen troughs containing the C_ferm_ diets without enzyme supplementation (186.6 and 156.0 ppm, respectively).

**Table 3:** Analysed chemical composition and biogenic amine concentrations of dietary treatments collected from the pen troughs at d10 (on an 88% DM basis, g/kg unless otherwise stated).

Cereal fermentation (C_ferm_)^a^	Fresh	Fresh	Ferm	Ferm	n	SD^c^
Enzyme (ENZ)^b^	**−**	** + **	**−**	+		
Gross energy, MJ/kg	16.1	16.2	15.9	16.1	4	0.76
Total oil	29.7	28.4	28.4	27.0	4	12.37
Crude fibre	34.5	30.9	36.0	32.4	4	4.59
Neutral detergent fibre	117.2	109.0	118.2	104.5	4	9.38
Acid detergent fibre	44.5	38.5	41.7	38.0	4	8.04
Ash	46.7	39.5	48.2	42.9	4	7.37
Crude protein	160.3	157.1	160.6	165.4	4	10.52
**Amino acids**						
Lysine	8.4	8.3	8.7	9.0	4	0.11
Methionine	2.6	2.6	2.6	2.7	4	0.02
Cysteine	2.8	2.7	2.6	2.8	4	0.02
Threonine	5.5	5.4	5.5	6.0	4	0.04
Tryptophan	2.0	2.1	1.9	2.3	4	0.02
Valine	7.0	6.8	7.0	7.3	4	0.06
**Biogenic amines**^**d**^**, ppm**						
Cadaverine	18.5	19.3	186.6	6.5	2	101.64
Tyramine	< 5	< 5	< 5	< 5	2	–
Putrescine	27.8	62.1	156.0	20.7	2	50.01
Spermine	< 5	7.3	< 5	< 5	2	2.04
Spermidine	34.9	31.2	31.6	36.0	2	5.57
Histamine	< 5	36.0	89.9	86.1	2	18.00
pH^d^	4.5	4.7	4.5	4.4	2	0.37
**Microbial counts**^**d,e**^**, CFU/g**						
Lactic acid bacteria	8.5	8.8	9.1	8.7	2	0.20
Enterobacteriaceae	5.5	5.6	5 .1	4.6	2	1.63
Yeasts	6.6	7.5	7.2	7.6	2	0.61
Moulds	< DL	< DL	< DL	< DL	2	–
**VFA concentration**^**d**^**, mmol/kg**						
Acetate	34.60	39.30	34.60	65.40	2	5.511
Propionate	0.42	0.55	0.56	0.65	2	0.242
Isobutyrate	0.92	1.00	0.77	0.94	2	0.099
Butyrate	0.32	0.40	0.28	0.32	2	0.109
Isovalerate	0.05	0.12	0.15	0.07	2	0.035
Valerate	0.22	0.20	0.16	0.30	2	0.057
Total VFA	36.52	41.57	36.51	67.69	2	5.448
Acetate:Propionate	88.24	96.68	69.30	167.82	2	51.023
Protein-derived VFA^f^	1.18	1.32	1.08	1.32	2	0.132

^a^Fermenting the cereal fraction of the diet prior to feeding (fresh = not fermented, ferm = fermented).

^b^Enzyme supplementation with a Xylanase and β-glucanase complex [unsupplemented (−), supplemented ( +)].

^*c*^*SD* standard deviation.

^d^On a fresh matter basis.

^e^*DL* detection limit (3 Log_10_ CFU/g).

^g^Protein-derived VFA calculated as the sum of isobutyrate, isovalerate and valerate.

### Impact of cereal fermentation and dietary enzyme supplementation on microbial diversity of the dietary components, dietary treatments and intestinal digesta of pigs

**Figure 1:**
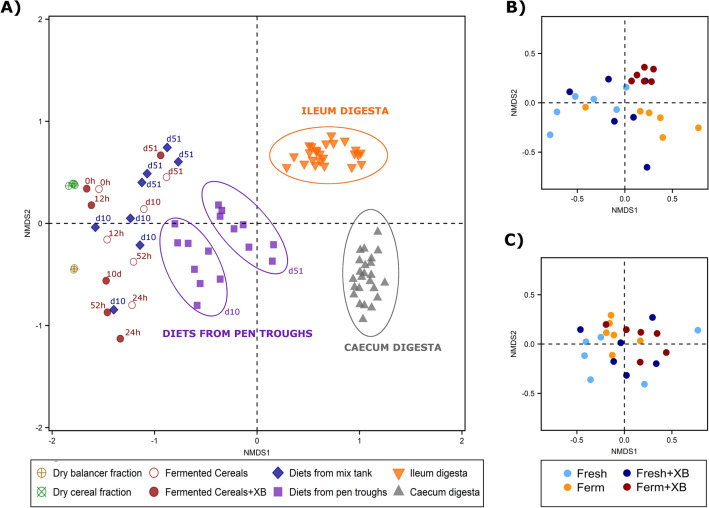
Non-metric multidimensional scaling plots showing the Bray–Curtis β-Diversity distance between sample type (**A**), ileal digesta samples (**B**) and caecal digesta samples (**C**).

No significant differences for any of the indices of α-diversity measured i.e. richness and evenness (Shannon and Simpson) were observed in the ileal and caecal digesta of pigs fed the dietary treatments (data not shown). The results of the β-diversity analysis are presented in Fig. 1. Clustering on the basis of sampling location (dry storage silos, fermentation tanks, mixing tanks, pen troughs, ileum and caecum) and sample type (dry dietary components, fermented cereals, diets and digesta) was observed, with dietary samples collected from the pen troughs distinctly different from ileal digesta samples, caecal digesta samples, and samples collected from the dry storage silos, fermentation tanks and mixing tanks (un-clustered) (Fig. 1A). Clustering on the basis of sampling time point was also observed for diets collected from the pen troughs (Fig. 1A). Partial clustering on the basis of dietary treatment was observed within the ileal digesta, with the digesta of pigs fed the C_ferm_ diets differing from that of pigs fed the fresh diets (Fig. 1B). No clustering on the basis of dietary treatment was observed within the caecal digesta (Fig. 1C).

### Impact of cereal fermentation and dietary enzyme supplementation on microbial composition of dietary components and dietary treatments

**Figure 2:**
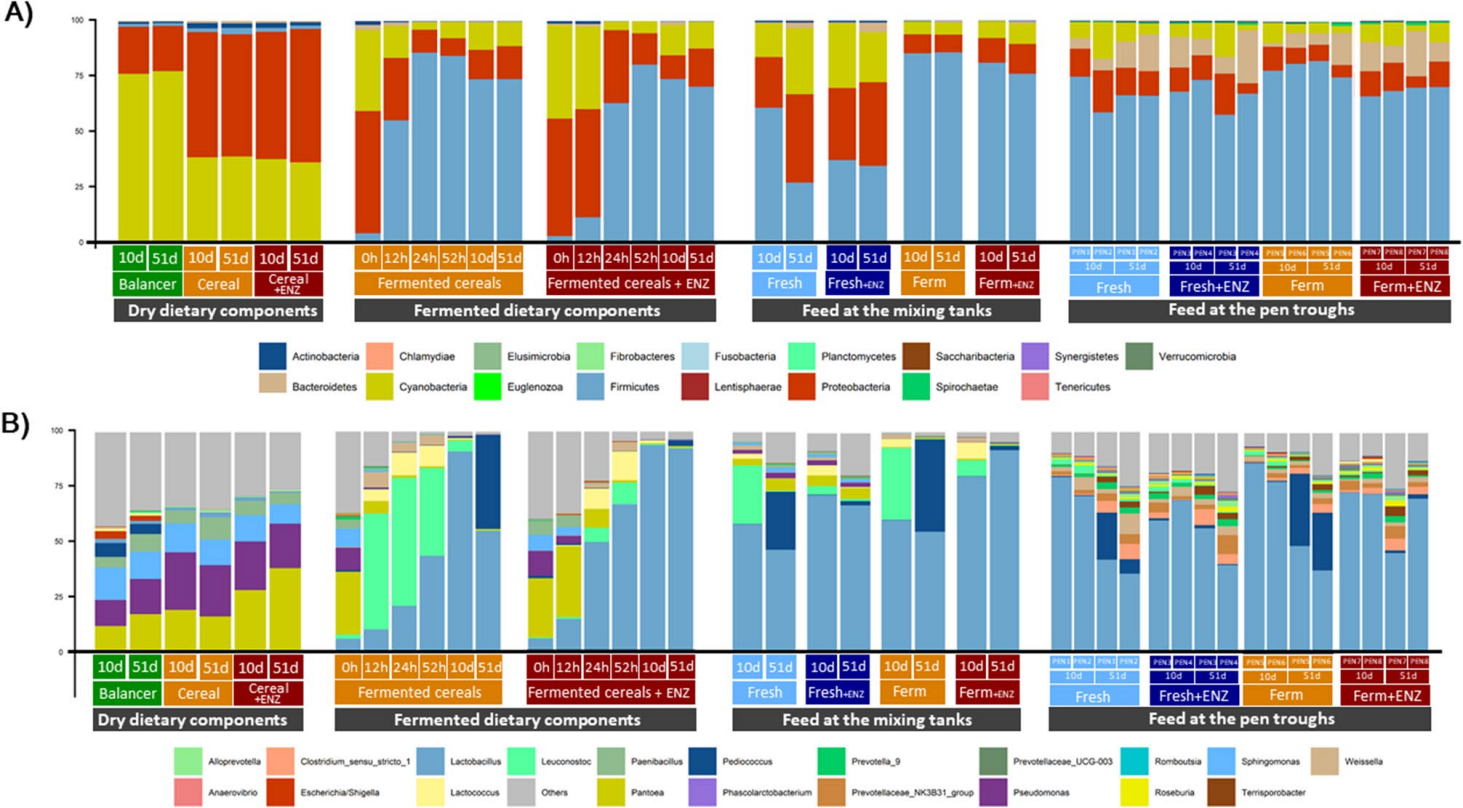
Relative abundance (%) of bacterial phyla (**A**) and the 20 most abundant bacterial genera (**B**) present in the dry dietary components (balancer, cereal and cereal + ENZ), the fermented dietary components (fermented cereals and fermented cereals + ENZ) at different time points (0 h, 12 h, 24 h and 52 h into the initial fermentation and 10d and 51d into the feeding trial), feed at the mixing tanks at different time points (d10 and d51) and feed collected at the pen troughs at different time points (d10 and d51).

Figure 2 shows the relative abundance (%) of bacterial phyla (Fig. 2A) and the 20 most abundant bacterial genera (Fig. 2B) present in the dry dietary components (BAL, CER and CER + ENZ), the fermented dietary components (fermented cereals and fermented cereals + ENZ) at different time points (0 h, 12 h, 24 h and 52 h into the initial fermentation and 10d and 51d into the feeding trial), as well as in feed sampled from the mixing tanks and pen troughs at d10 and d51. The most abundant phyla in the dry dietary components were *Proteobacteria* and *Cyanobacteria*, with the latter predominating in the BAL and the opposite occurring in the CER, and at genus level the dominant genera were *Pantoea* (21.6%) and *Pseudomonas* (20.0%). When the CER and CER + ENZ were mixed with water in the fermentation tanks (time 0 h), the microbial profile was similar to that found in the dry CER and CER + ENZ. At the end of the initial 52 h fermentation, the phylum *Firmicutes* (82.4%) and the genus *Lactobacillus* (52.2%) dominated the fermentation tanks, which was still the case at d10 of the feeding trial (73.8% and 92.4% relative abundance, respectively). However, at d51, while the fermented CER + ENZ was still dominated by *Lactobacillus* (92.3%), two genera predominated in the fermented CER tank [*Lactobacillus* (54.9%) and *Pediococcus* (42.6%)]. In the mixing tanks, the fresh liquid diets (Fresh and Fresh + ENZ) had a lower abundance of *Firmicutes* (39.9 vs. 82.1%, SD 14.6, n = 4) and a higher abundance of *Proteobacteria* (33.2 vs. 10.2%, SD 2.44, n = 4) than the fermented diets (Ferm and Ferm + ENZ). In the mixing tanks at d10, the un-supplemented diets were dominated at the genus level by *Lactobacillus* (58.8%) and *Leuconostoc* (29.8%), while the ENZ-supplemented diets were mainly dominated by *Lactobacillus* (75.8%). However, at d51 in the mixing tanks, the un-supplemented diets were dominated by *Lactobacillus* (50.4%) and *Pediococcus* (34.4%), while *Lactobacillus* still predominated in the ENZ-supplemented diets (79.4%). In the diets collected at the troughs, no differences in the microbial composition were observed between dietary treatments at phylum (Fig. 2A) or genus (Fig. 2B) level.

### Impact of cereal fermentation and dietary enzyme supplementation on nutrient digestibility, intestinal pH and intestinal VFA concentrations

Results for AiD and ATTD of DM, organic matter, CP, GE, and the dietary digestible energy (DE) are presented in Table 4. No interaction between C_ferm_ and ENZ supplementation was observed for AiD and ATTD for any of the nutrients analysed. Supplementation of ENZ to pig diets improved AiD and ATTD of DM, OM, CP and GE. Fermentation of the cereal fraction of pig diets improved ATTD of DM, OM, and CP but not their AiD. The ATTD of GE and the dietary DE value were improved when pigs were fed the C_ferm_ and ENZ-supplemented diets. The pH, and VFA concentrations in the ileal and caecal digesta of pigs are presented in Table 4. No interaction between C_ferm_ and ENZ supplementation was observed for the pH of the ileal digesta; however, the pH was higher in the ileal digesta of pigs fed the C_ferm_ diets compared to that of pigs fed the fresh diets (6.06 vs. 5.65, P < 0.05). There was an interaction between C_ferm_ and ENZ supplementation for the pH of the caecal digesta (P < 0.05). The ENZ supplementation increased the caecal pH of pigs fed the C_ferm_ diets but it did not increase the pH of those fed the fresh diets. There was no C_ferm_ * ENZ interaction or main effect of C_ferm_ or ENZ on total VFA concentrations (Table 4) or on the concentrations of individual VFAs in the ileum (data not shown). In the caecum, supplementation with ENZ reduced the concentration of total VFA and propionate and increased the concentration of isobutyrate, while C_ferm_ reduced concentrations of propionate and butyrate also in the caecum.

**Table 4:** Effect of dietary fermentation of cereals with or without carbohydrase supplementation on apparent ileal digestibility (AiD), apparent total tract digestibility (ATTD), pH and volatile fatty acid (VFA) concentrations in the ileal and caecal digesta of grow-finisher pigs (n = 6)^1^.

Cereal fermentation (C_ferm_)^2^:		Fresh	Fresh	Ferm	Ferm		P-value			
Enzyme (ENZ)^3^:		**−**	** + **	**−**	** + **	SEM^4^	ENZ	C_ferm_	ENZ*C_ferm_	
**AiD**										
Dry matter	65.22		69.19	63.17	67.01	1.632	0.03	0.21		0.97
Organic matter	64.24		68.33	61.81	68.33	2.060	0.02	0.19		0.93
Crude protein	41.19		37.31	51.10	44.69	4.015	0.02	0.16		0.72
Gross energy	63.02		68.82	61.95	63.82	1.740	0.02	0.07		0.20
**ATTD**										
Dry matter	83.23		85.41	84.79	85.98	0.286	< 0.001	< 0.01		0.08
Organic matter	84.28		86.49	85.73	86.92	0.327	< 0.001	< 0.05		0.10
Crude protein	80.68		84.25	84.44	85.94	0.635	< 0.001	< 0.01		0.13
Gross energy	82.24		84.77	83.84	85.55	0.388	< 0.001	< 0.01		0.23
Digestible energy, MJ/kg	14.91		15.43	15.25	15.61	0.071	< 0.001	< 0.001		0.18
**Ileal digesta**										
pH	5.61		5.69	5.93	6.18	0.184	0.36	< 0.05		0.61
Total VFA^5^	71.24		73.12	72.50	58.86	7.302	0.37	0.42		0.29
**Caecal digesta**										
pH	5.27^b^		5.27^b^	5.24^b^	5.66^a^	0.063	< 0.01	< 0.01		< 0.01
Acetate	129.54		133.70	137.34	119.25	6.504	0.26	0.62		0.10
Propionate	42.31		36.19	36.01	31.09	2.303	< 0.05	< 0.05		0.75
Isobutyrate	0.87		1.01	0.80	1.08	0.102	< 0.05	0.98		0.47
Butyrate	21.12		21.29	18.92	15.20	1.315	0.09	< 0.01		0.06
Isovalerate	1.36		1.51	1.30	1.52	0.133	0.17	0.82		0.77
Valerate	2.95		2.20	1.79	1.90	0.365	0.36	0.05		0.24
Total VFA	196.81		194.20	195.40	168.69	7.120	< 0.05	0.07		0.10
Acetate:Propionate	3.25		3.76	3.93	3.91	0.251	0.28	0.07		0.23
Protein-derived VFA^6^	5.18		4.71	3.91	4.48	0.503	0.91	0.12		0.28

^1^Values within a row that do not share a common superscript are statistically different (P < 0.05).

^2^Fermenting the cereal fraction of the diet prior to feeding (fresh = not fermented, ferm = fermented).

^3^Enzyme supplementation with a Xylanase and β-glucanase complex [unsupplemented (−), supplemented ( +)].

^4^*SEM* standard error of the mean.

^5^Individual concentrations of ileal VFA were not significantly different and therefore only the total VFA concentration is shown.

^6^Protein-derived VFA calculated as the sum of isobutyrate, isovalerate and valerate.

### Impact of cereal fermentation and enzyme dietary supplementation on pig growth and carcass quality traits

Pig growth and carcass quality traits are presented in Table 5. An interaction between C_ferm_ and ENZ supplementation was observed for LW at d28 (P < 0.05) and d55 (P < 0.001). Pig LW was increased when the un-supplemented cereal was fermented prior to feeding, whereas LW remained unchanged when the ENZ-supplemented cereal was fermented. No interactions were found for any of the other growth or carcass quality traits. The ADG of pigs fed the C_ferm_ was 4.1% higher than that of pigs fed the fresh diets (P < 0.01), whereas the FCR of pigs fed the ENZ-supplemented diets was improved (3.8% lower, P < 0.05). Pigs fed the C_ferm_ diets had 2.0 kg heavier carcasses (P < 0.01) and 1.0% lower lean meat percentage at slaughter (P < 0.05). There was no effect of sex or its interactions with C_ferm_ or ENZ for any of the growth parameters measured (live weight, ADG, ADFI or FCR), the effect of sex and its interactions with C_ferm_ and ENZ on carcass quality are reported in supplementary Table S1 online.

**Table 5:** Effect of dietary cereal fermentation with or without carbohydrase supplementation on growth, feed intake, feed efficiency and carcass quality of grow-finisher pigs (n = 9)^1^.

Cereal fermentation (C_ferm_)^2^:	Fresh	Fresh	Ferm	Ferm		P-value		
Enzyme (ENZ)^*3*^*:*	**−**	** + **	**−**	+	SEM^4^	ENZ	C_ferm_	ENZ*C_ferm_
**Live weight, kg**								
Day 0	40.7	40.6	40.6	40.7	0.50	0.99	0.92	0.99
Day 14	49.1	50.1	49.8	50.1	0.50	0.21	0.45	0.47
Day 28	66.1^c^	66.8^b,c^	68.2^a^	67.7^a,b^	0.50	0.92	< 0.01	< 0.05
Day 55	95.1^c^	96.8^a,b^	98.1^a^	97.5^a,b^	0.50	0.23	< 0.01	< 0.001
**Growth**								
ADG^5^, g/d	963	993	1,024	1,012	13.2	0.48	< 0.01	0.08
ADFI^6^, g/d	2,608	2,583	2,659	2,594	35.5	0.20	0.37	0.56
FCR^7^, g/g	2.81	2.67	2.71	2.65	0.047	< 0.05	0.17	0.45
**Carcass quality**								
Carcass weight, kg	71.8	72.8	74.5	74.1	0.61	0.62	< 0.01	0.22
Kill out, %	76.1	75.7	76.1	76.4	0.23	0.77	0.09	0.11
Muscle depth, mm	47.5	49.0	47.5	47.2	0.46	0.18	0.06	0.06
Fat depth, mm	11.2	11.9	11.9	12.1	0.27	0.12	0.11	0.40
Lean meat %	57.8	57.5	57.2	57.0	0.24	0.25	< 0.05	0.79

^1^Values that do not share a common superscript are statistically different (P < 0.05).

^2^Fermenting the cereal fraction of the diet prior to feeding (fresh = not fermented, ferm = fermented).

^3^Enzyme supplementation with a xylanase and β-glucanase complex [unsupplemented (−), supplemented ( +)].

^4^*SEM* standard error of the mean.

^5^*ADG* average daily gain.

^*6*^*ADFI* Average daily feed intake.

^7^*FCR* feed conversion ratio.

### Impact of cereal fermentation and dietary enzyme supplementation on the intestinal microbiota composition of pigs

**Figure 3:**
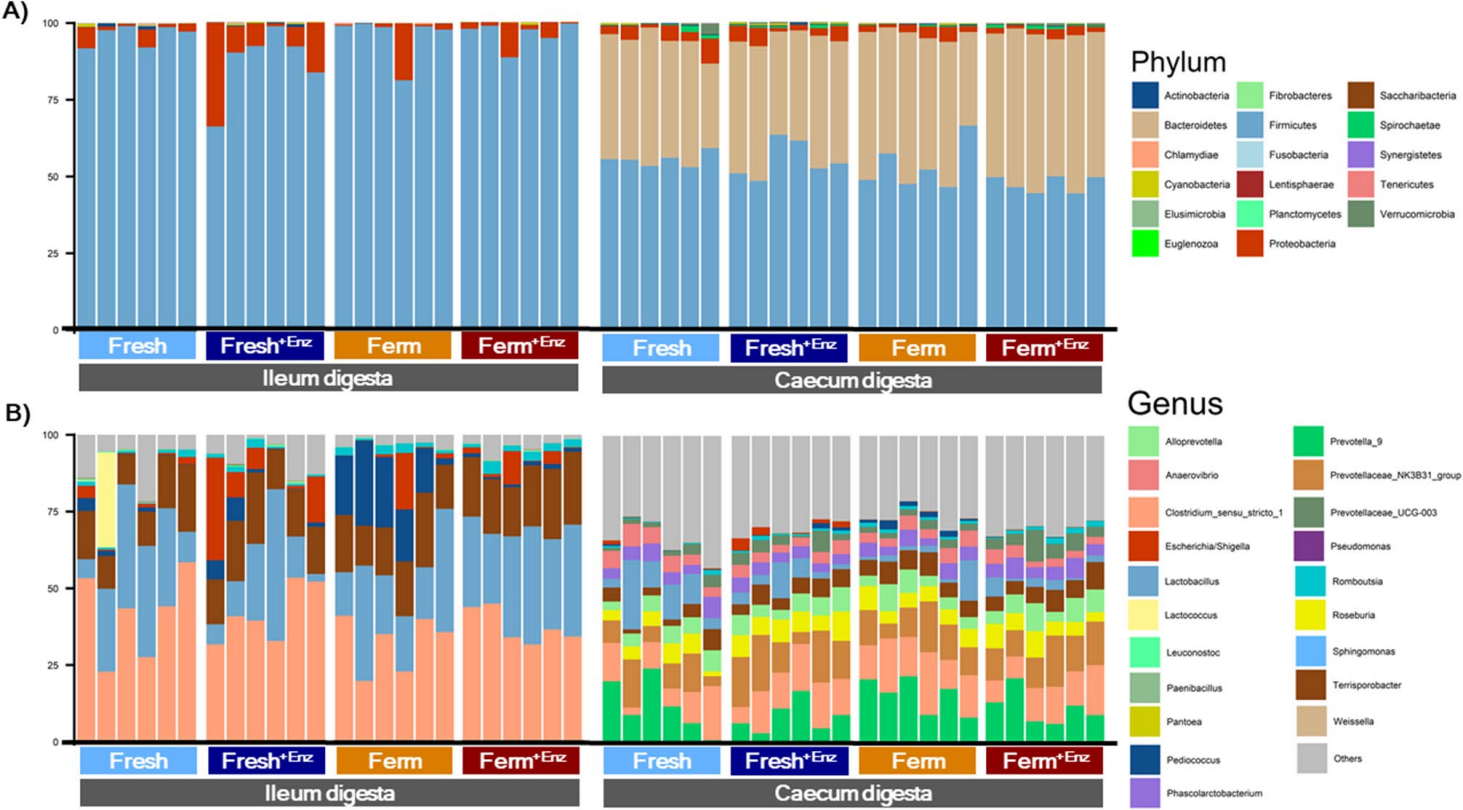
Relative abundance (%) of bacterial phyla (**A**) and the 20 most abundant bacterial genera (**B**) present in the ileum and caecum digesta of pigs fed the experimental dietary treatments. Each bar represents the bacterial profile of the corresponding sample for each individual pig (n = 6/treatment). ^1^Others = All bacterial genera not included in the 20 most abundant.

The intestinal microbial composition at phylum and genus level is presented in Fig. 3. Figure 3A presents the relative abundance (%) of bacterial phyla in the ileum and caecum of pigs fed the different dietary treatments. *Firmicutes* (93.8%) and *Proteobacteria* (5.7%) were the two most abundant phyla in the ileum while *Firmicutes* (52.7%) and *Bacteroidetes* (42.8%) predominated in the caecal digesta. Figure 3B presents the relative abundance (%) of the 20 most abundant bacterial genera observed in the ileum and caecum of pigs fed the different diets. *Clostridium_senso_stricto* (38.3%) was the most abundant genus in the ileal digesta, whereas *Prevotella_9* (11.9%) and *Clostridium_senso_stricto* (11.7%) were the most abundant in the caecal digesta (Fig. 3B).

**Figure 4:**
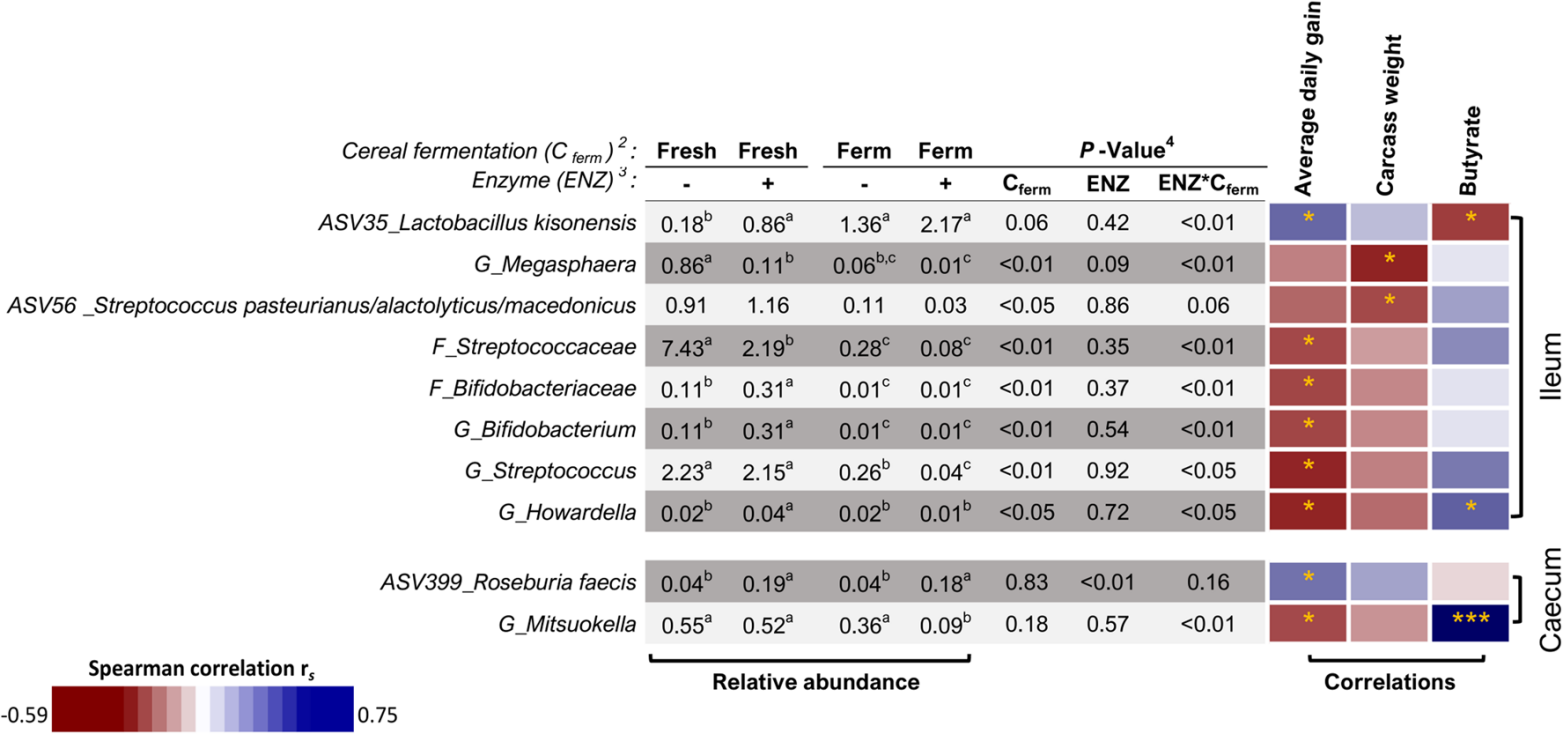
Heatmap showing Spearman correlations between the ileal and caecal bacterial taxa found to be differentially abundant between dietary treatments and selected physiological measures in pigs fed the different dietary treatments (n = 6/treatment). Taxa are reported at genus (G) and exact amplicon sequence variant (ASV) level. The median relative abundance of each taxon for each dietary treatment is reported in the adjacent table. Positive correlations are indicated in blue and negative correlations are indicated in red. Significant correlations are indicated with asterisks (P < 0.05 = *, P < 0.01 = **)^1^. ^1^Within a row, values that do not share a common superscript are statistically different (P < 0.05). ^2^Fermenting the cereal fraction of the diet prior to feeding (fresh = 0 h fermentation, Ferm = 52 h initial fermentation). ^3^Enzyme supplementation with a xylanase and β-glucanase complex [unsupplemented (−), supplemented ( +)]. ^4^P-value corrected for false discovery rate (FDR).

The relative abundance of phyla and genera that were differentially abundant (P < 0.05) in response to dietary treatment (effect of ENZ, C_ferm_, or ENZ*C_ferm_ interaction) in the ileal and caecal digesta are presented in Table 6, and the differentially abundant families and exact ASV are presented in supplementary Table S2 online. Differences between dietary treatments were observed in the ileum for 4 phyla, 5 families, 19 genera and 35 ASV. The most striking difference was the interaction between C_ferm_ and ENZ for the relative abundance of *Pediococcus* in the ileum. The ileal abundance of *Pediococcus* was higher when pigs were fed diets containing un-supplemented cereals which were fermented prior to feeding but it remained unchanged in pigs fed the diet containing fermented ENZ-supplemented cereals. In the caecum, 4 families, 12 genera and 14 ASV were differentially abundant due to treatment. Spearman correlations between the differentially abundant taxa and all recorded physiological measures in the ileum and caecum of pigs are presented in Figure S2 and Figure S3 online, respectively. From this, the differentially abundant taxa that were significantly correlated with ADG, carcass weight and/or intestinal butyrate concentration are extracted and presented in Fig. 4. *Lactobacillus kisonensis* (ASV35) was more abundant in the ileum when the pigs were fed the ENZ-supplemented diets, the fermented diets or both and *Roseburia faecis* (ASV399) had higher caecal abundance when pigs were fed the ENZ-supplemented diets. Both taxa were positively correlated with ADG. The ileal abundance of *G_Megasphaera, Streptococcus pasteurianus/alactolyticus/macedonicus* (ASV56), *G_Bifidobacterium, G_Streptococcus* and *G_Howardella* were negatively correlated with ADG or carcass weight. An interaction between C_ferm_ and ENZ supplementation was found for the relative abundance of the aforementioned ileal taxa. C_ferm_ reduced their ileal abundance but ENZ supplementation increased/reduced their ileal abundance only when supplemented to fresh diets (i.e. *G_Megasphaera, G_Bifidobacterium* and *G_Howardella*) or only when supplemented to fermented diets (i.e. *G_Streptococcus*). In the case of *G_Megasphaera,* ENZ supplementation reduced its ileal abundance when the fresh diet was ENZ-supplemented but not when the fermented diet was ENZ-supplemented. In the case of *G_Bifidobacterium* and *G_Howardella*, ENZ supplementation increased their ileal abundance when the fresh diet was ENZ-supplemented but not when the fermented diet was ENZ-supplemented. In the case of *G_Streptococcus*, ENZ supplementation increased its ileal abundance when the fermented diet was supplemented but not when the fresh diet was supplemented.

**Table 6:** Relative abundance (%) of microbial taxa differentially abundant according to dietary treatment in the ileal and caecal digesta of pigs^1^.

Cereal fermentation (C_ferm_)^2^:	Fresh	Fresh	Ferm	Ferm		P-value^5^		
Enzyme (ENZ)^3^:	−	+	−	+	SEM^4^	ENZ	C_ferm_	ENZ*C_ferm_
**Ileum**^**6**^								
P_Bacteroidetes	0.22^a^	0.23^a^	0.03^b^	0.06^a,b^	0.104	0.76	< 0.01	< 0.05
P_Tenericutes	0.01^b^	0.00^b^	0.19^a^	0.04^a,b^	0.098	0.33	< 0.01	< 0.05
P_Chlamydiae	0.10^a^	0.01^a,b^	0.05^a^	0.00^b^	0.064	0.08	0.42	< 0.05
P_Actinobacteria	0.43^a^	0.28^a^	0.05^b^	0.02^b^	0.185	0.58	< 0.001	< 0.01
G_Romboutsia	1.08^b^	1.30^b^	1.95^a,b^	2.56^a^	0.573	0.73	< 0.05	0.11
G_Stenotrophomonas	0.08^a^	0.10^a^	0.03^a,b^	0.01^b^	0.059	0.82	< 0.05	0.11
G_Enterococcus	1.83^a^	0.94^a^	0.05^a,b^	0.01^b^	2.531	0.95	< 0.05	0.10
G_Brucella	0.02^a^	0.02^a^	0.01^a,b^	0.01^b^	0.006	0.75	< 0.05	0.10
G_Phascolarctobacterium	0.06^a^	0.01^a,b^	0.01^a,b^	0.00^b^	0.029	0.17	< 0.05	0.09
G_Alloprevotella	0.04^a^	0.02^a,b^	0.00^c^	0.01^c,b^	0.019	0.76	< 0.05	0.07
G_Mitsuokella	0.07^b^	0.65^a^	0.03^b^	0.01^b^	0.436	0.43	< 0.05	< 0.05
G_Chlamydia	0.10^a^	0.01^a,b^	0.05^a^	0.00^b^	0.064	0.10	0.49	< 0.05
G_Actinobacillus	0.78^a^	1.00^a^	0.09^a,b^	0.02^b^	0.616	0.98	< 0.01	< 0.05
G_Aerococcus	0.01^a^	0.01^b^	0.00^d^	0.00^c^	0.000	0.95	0.11	< 0.05
G_Howardella	0.02^b^	0.04^a^	0.02^b^	0.01^b^	0.008	0.72	< 0.05	< 0.05
G_Campylobacter	0.02^c^	0.03^c,b^	0.07^a,b^	0.20^a^	0.086	0.61	< 0.01	< 0.05
G_Mycoplasma	0.01^b^	0.00^b^	0.19^a^	0.05^a^	0.085	0.73	< 0.01	< 0.05
G_Veillonella	1.65^a^	0.69^a^	0.59^a^	0.07^b^	0.796	0.43	< 0.05	< 0.01
G_Megasphaera	0.86^a^	0.11^b^	0.06^b,c^	0.01^c^	0.424	0.09	< 0.01	< 0.01
G_Bifidobacterium	0.11^a^	0.31^a^	0.01^b^	0.01^b^	0.147	0.54	< 0.001	< 0.01
G_Lactococcus	6.28^a^	0.07^b^	0.02^b^	0.06^b^	4.257	< 0.05	< 0.01	< 0.01
G_Pediococcus	1.28^b^	2.79^b^	17.27^a^	1.12^b^	6.533	0.09	< 0.05	< 0.01
G_Streptococcus	2.23^a^	2.15^a^	0.26^b^	0.04^c^	0.932	0.92	< 0.01	< 0.01
**Caecum**^**6**^								
G_Coprococcus_1	0.32^a^	0.13^b^	0.12^b^	0.25^a^	0.064	0.70	0.76	< 0.05
G_Candidatus_Soleaferrea	0.06^a^	0.01^b^	0.01^b^	0.01^b^	0.016	0.32	0.20	< 0.05
G_Defluviitaleaceae_UCG-011	0.06^a^	0.00^c^	0.01^b^	0.01^b^	0.009	0.32	0.41	< 0.05
G_Lachnospiraceae_NK4B4_group	0.02^c^	0.07^a,b^	0.03^cb^	0.12^a^	0.027	0.09	0.35	< 0.05
G_Ruminococcaceae_UCG-002	0.11^a^	0.02^b^	0.02^b^	0.02^b^	0.030	0.13	0.18	< 0.05
G_Selenomonas_3	0.13^a^	0.11^a^	0.12^a^	0.01^b^	0.050	0.53	0.61	< 0.01
G_Intestinibacter	0.32^a^	0.37^a^	0.28^a^	0.15^b^	0.051	0.64	0.18	< 0.01
G_Mitsuokella	0.55^a^	0.52^a^	0.36^a^	0.09^b^	0.154	0.57	0.18	< 0.01
G_Pediococcus	0.17^c,b^	0.51^a,b^	1.63^a^	0.10^c^	0.656	0.32	0.35	< 0.01
G_Streptococcus	0.79^a^	1.03^a^	0.11^b^	0.01^c^	0.512	0.93	0.02	< 0.01
G_Megasphaera	3.22^a^	1.18^a^	1.01^a^	0.01^b^	1.599	0.45	0.30	< 0.01
G_Escherichia/Shigella	0.34^b^	1.73^a^	0.08^c^	0.11^b,c^	0.679	0.24	0.01	< 0.01

^1^Values within a row that do not share a common superscript are statistically different (P < 0.05).

^2^Fermenting the cereal fraction of the diet prior to feeding (fresh = un-fermented, ferm = fermented).

^3^Enzyme supplementation with a xylanase and β-glucanase complex [unsupplemented (−), supplemented ( +)].

^4^*SEM* standard error of the mean.

^5^P-value corrected for false discovery rate (FDR).

^6^*P_* = Phylum, *G_* = Genus.

## Discussion

Cereals were successfully fermented after the initial 52 h of fermentation using a *Lactobacillus/Pediococcus* inoculum and all parameters measured were as expected: pH was low (3.7), LAB counts were high (9.2 log_10_ CFU/g), *Enterobacteriaceae* were undetectable, yeast counts were 5.8 log_10_ CFU/g and total VFA concentrations were 24–29 mmol/g. These results are in line with values reported in other studies where wheat, barley or wheat distillers dry grains with solubles were fermented under similar conditions^11,13,39,40^. Bacterial profiling data, generated using 16S rRNA gene sequencing, revealed that after an initial 52 h of fermentation set-up, the relative abundance profile at genus level had shifted from one in which *Pantoea* and *Pseudomonas* (species of which are opportunistic pathogens) predominated, to one in which LAB were dominant i.e. *Lactobacillus* predominated, followed by *Leuconostoc* and *Lactococcus*. On d10 of the feeding experiment, *Lactobacillus* was still the predominant genus in both fermentation tanks. However, towards the end of the experiment (d51) *Pediococcus parvulus* and *Lactobacillus* (mainly *L. paralimentarius*, *L. silagincola, L. buchneri, L. zymae, L. kisonensis* and *L. plantarum*) dominated the un-supplemented cereals, but only *Lactobacillus* dominated the ENZ-supplemented cereals (mainly *L. farciminis, L. parlamentarius, L. rossiae, L. paralimentaris* and *L. kisonensis*). This is in agreement with the fact that *P. parvulus* is unable to utilise xylose, one of the polysaccharides released by the ENZ complex^41^ (the other one being β-glucans) while many *Lactobacillus* species can^42^. The *L. plantarum* and *P. acidilactici* used to inoculate the cereal at the beginning of the fermentation set-up did not dominate the fermentation tanks, as would perhaps have been expected; the former was found in the fermentation tanks at time 52 h at a relative abundance < 2% and the latter was not detected. In order to confirm this lack of predominance of the inoculum strains, the identity of the species contained within the inoculum was confirmed by 16S rRNA gene sequencing (data not shown). In the mixing tanks, the diets containing fermented cereals had a higher abundance of *Lactobacillus* and lower abundance of *Proteobacteria* than the fresh diets. While to our knowledge, this is the first study to report the full microbial profile of fermented cereals or fermented liquid diets, these results are in agreement with studies showing a higher concentration of LAB when cereals are fermented^13,38–40,43^. Despite seeing differences in bacterial profile between diets in the mixing tanks, the differences in bacterial profile between dietary treatments were less obvious when diets were collected from feed troughs in the pig pens, indicating that some degree of spontaneous fermentation had occurred within the troughs. The pH, microbial counts and VFA concentrations found in C_ferm_ diets were in line with the standard values for regular liquid feed reported by SEGES, Danish Pig Research Centre^44^. However, the pH and acetate concentration in Fresh diets was slightly above the standard values reported in fresh liquid diets^44^. High acetate concentration in liquid feed is associated with reduced palatability^45^; however, in the current experiment feed refusal was not observed. We also found evidence of spontaneous fermentation in the troughs with this type of liquid feeding system when fresh liquid diets were fed^31^.

No impact of dietary treatments on the main macronutrients analysed (CP, oil, ash, CF, NDF, ADF or GE) in samples collected at the trough was observed. This is in line with the findings of Moran et al.^13^ who observed no differences in CP, oil and ash when wheat was fermented for 24 h. Because of the fermentation process (which is also likely occurring in the feed troughs), bacterial degradation of AA (particularly free AA) can potentially occur, with the resultant formation of biogenic amines^11,13,45,46^. Cadaverine and putrescine are biogenic amines formed by the decarboxylation of lysine^47,48^. In this study, only the cereal fraction of the diet was fermented in order to minimise the time that synthetic AA (included in the balancer fraction) were in contact with water prior to feeding. This strategy appears effective, as only minimal degradation of dietary lysine was observed (reductions from the mixing tank to the trough of 12% and < 9% were observed for the fresh and cereal-fermented diets, respectively). Even with spontaneous fermentation in the troughs, the concentrations of biogenic amines were low/comparable to the levels normally found in liquid feed on farms. The maximum concentrations of cadaverine and putrescine observed in the current study were 186 and 156 ppm, respectively, while Le Treut^49^, in a study comprising samples from 33 French liquid-fed farms, reported averages of 192 and 70 ppm, respectively, with maximum levels as high as 1,182 and 310 ppm. In the current experiment, feed refusal was not observed, indicating that the levels of cadaverine and putrescine in the diet had little if any impact on feed acceptance. Despite the well-known toxic effects of high concentrations of biogenic amines in feed and food^50,51^, we failed to find clear guidelines or regulations regarding the maximum acceptable levels in liquid pig feed. Moreover, our results are in line with those from another study where only the cereal fraction of the diet was fermented and the concentrations of cadaverine and putrescine were 153 and 75 ppm, respectively^52^.

Improvements in pig growth and nutrient digestibility due to cereal fermentation were observed in this study, with increased ADG, and ATTD of DM, OM, CP and GE found in grow-finisher pigs. In agreement with our results, cereal fermentation and cereal soaking increased the ATTD but not the AID of liquid diets in previous studies^39,44^. This suggests that feeding fermented cereals to the pig has a greater impact in the large intestine than in the small intestine, probably due to changes in the metabolites produced by the bacterial population in the large intestine. For instance, in the current study no changes in VFA concentrations were observed in the ileum, while cereal fermentation reduced the concentrations of propionate and butyrate in the caecum. This may be because complete fermentation of fermentable carbohydrates in the diet had likely occurred during the fermentation of cereals prior to feeding, thereby depleting the substrate available for fermentation in the lower GIT. Further to this, butyrate and propionate produced by this cereal pre-fermentation and from any further fermentation of the diet during feeding would largely be used in the upper GIT (i.e. absorption, epithelial cell proliferation, gut barrier improvement, etc.), leaving little to reach the large intestine^53,54^. The current study found that ADG was increased when pigs were fed the cereal-fermented diets and this is contrary to the results from previous studies where no improvements, or even decreased growth, were found^11,51,52^. However, the latter studies were all conducted with younger pigs (weaned piglets) where the GIT is not fully developed, cereal-fermented diets were compared to dry pelleted feed and the feed in these experiments was not offered ad-libitum. These differences help to explain why results from the current study are not consistent with those previously reported. Supplementation of a xylanase and β-glucanase complex to the diet increased DM, OM, CP and GE AiD and ATTD, which consequently improved the G:F of grow-finisher-pigs in the current study. In agreement with our results, Jakobsen et al.^55,56^ observed improvements in AiD and ATTD when a complex of enzymes containing xylanase and β-glucanase was supplemented to fermented by-products fed to grow-finisher pigs; however, growth performance data were not reported in these studies. Other studies, found no effects of enzyme supplementation to liquid, soaked and fermented liquid diets on nutrient digestibility, growth or FE^43,57–61^.

A recent meta-analysis defining the core microbiota in the GIT of pigs^62^ reported similar intestinal microbiota composition to that observed in the current study, in that the three most abundant phyla in the ileal and caecal digesta were the same (*Firmicutes*, *Proteobacteria* and *Bacteroidetes*). Some differences at genus level between the defined core microbiota profile^62^ and the microbiota profile in our study were observed. These differences might be partially explained by the fact that this is, to our knowledge, the first study reporting the intestinal microbiota profile of pigs fed cereal-fermented liquid feed while the studies included in the meta-analysis were conducted using pigs fed diets in dry form. The most obvious difference observed is the high abundance of *Pediococcus parvulus* in the ileal digesta in the present study (mainly in pigs fed the fermented cereal diet without enzyme). Among other differences, in the ileum, *Lactobacillus, Clostridium* and *Terrisporobacter* were the three most abundant genera in our study, while the three most abundant genera reported in the afore-mentioned meta-analysis were *Lactobacillus, Clostridium* and *Streptococcus*. In the caecum, *Prevotella, Clostridium* and *Prevotellaceae*_NK3B31_group were the three most abundant genera in our study, while *Prevotella, Escherichia/Shigella* and *Clostridium* were the three most abundant genera in the afore-mentioned meta-analysis.

As regards the effect of cereal fermentation and/or dietary enzyme supplementation on the intestinal microbiome, the relative abundance of *L. kisonensis* was increased in the ileum of pigs fed the fresh liquid diets supplemented with enzyme and tended to increase when the diets were fermented (0.61 vs. 1.8%, P = 0.06). It is possible that *L. kisonensis* benefited from oligosaccharides released by the enzyme and the fermentation process. In fact, it is capable of utilizing xylose^63^ which is released by xylanase. Ileal abundance of *Lactobacillus kisonensis* (ASV35) was positively correlated with ADG and negatively correlated with ileal butyrate concentration. We cannot find an explanation for the negative correlation with butyrate but as regards the positive correlation with ADG, lactobacilli have been used as probiotics in swine for many years and are associated with lower counts of pathogenic bacteria in the GIT^64,65^. In agreement with the findings from the current study, the *Lactobacillus* genus and different operational taxonomic units belonging to *Lactobacillus* have previously been shown to be more abundant in pigs with improved FE^6,9^, although no association with FE was found in the current study. To our knowledge, this is the first study showing an association between *L. kisonensis* and pig growth. Again, we have no definitive explanation for this but, as regards metabolites produced by this species, it is heterofermentative and hence ferments glucose into compounds including ethanol, acetic acid and CO_2_, in addition to the lactic acid produced by homofermentative species. Ethanol and acetic acid have antimicrobial effects which would be additive to the effect of lactic acid. Apart from reducing pathogen load, *Lactobacillus* also produce a range of enzymes which can break down feed ingredients^66^, thereby yielding additional energy for the animal. However, *L. kisonensis* appears to have a relatively narrow fermentative capability^63^.

Improvements in pig growth and nutrient digestibility due to cereal fermentation were observed in the current study. Dietary enzyme supplementation increased the AiD and ATTD of the diets and as a consequence the G:F of grow-finisher-pigs was improved. Ileal abundance of *Megasphaera*, *Bifidobacterium*, *Streptococcus*, *Howardella* and *Streptococcus pasteurianus/alactolyticus/macedonicus* (ASV56) were negatively correlated with either carcass weight or pig growth and all were lower in abundance in pigs fed fermented cereal diets, possibly explaining the better growth in these animals. To the author´s knowledge, associations between carcass weight and intestinal bacterial composition have not previously been reported. In agreement with our results, *Streptococcus* was previously found to be more abundant in pigs with poorer FE^8^. It is possible that the oligosaccharides released by cereal fermentation favoured the growth of other genera, thereby excluding streptococci. Contrary to our results, the *Megasphaera* genus was more abundant in the ileum and caecum of pigs with improved FE in a previous study^9^ in which pigs were fed a dry corn-based diet. In a recent study of ours also performed with liquid-fed pigs using the same facilities as in the current study^31^ the caecal abundance of *Megasphaera elsdenii* was positively correlated with pig growth and its relative abundance was lower in pigs fed fresh liquid diets supplemented with the same ENZ complex. In the current study, the abundance of the *Megasphaera* genus tended to be reduced when the ENZ complex was supplemented to the diets, but in the ileum. *Megasphaera* species are unable to metabolize xylose^67^ (the product of xylanase activity); therefore, it can be speculated that supplementation of the ENZ complex in the current study favoured the growth of microbial taxa more adapted to xylose utilisation in the ileum. To our knowledge, associations between pig growth parameters and ileal abundance of *Bifidobacterium* and *Howardella* have not previously been reported in the literature. We cannot find a direct reasoning for their negative correlation with pig growth, nor for the positive correlation between *Howardella* and ileal butyrate concentration. In the current study, caecal abundance of *Roseburia faecis/intestinalis* (ASV399) was positively correlated with pig growth and its relative abundance was higher in pigs fed the enzyme-supplemented diets, possibly helping to explain the improved FE in these animals. In agreement with this, the caecal abundance of *Roseburia hominis, intestinalis* and *inulinivorans* has previously been associated with improved FE in pigs^9^. *Roseburia faecis* and *intestinalis* are associated with butyrate production in the hindgut of pigs^68^. In spite of this, in the current study, their abundance was not correlated with butyrate concentration in the caecum. However, analysis of colon samples may reveal otherwise and colon samples were not taken in the current study. Caecal abundance of the *Mitsuokella* genus was negatively correlated with pig growth and caecal butyrate concentration in the current study and its abundance was reduced in pigs fed the cereal-fermented diet supplemented with enzymes. We cannot find an explanation for this negative correlation; however, contrary to these findings, the caecal abundance of *Mitsuokella* has previously been found to be higher in pigs with improved FE, but when fed a dry corn-based diet^9^.

Overall, in the current study, the two bacterial taxa (*L. kisonensis* and *R. faecis*) that were positively correlated with pig growth were more abundant in the ileum/caecum of pigs fed the enzyme-supplemented diets (only for fresh liquid diets in the case of *L. kisonensis*). Therefore, exogenous enzyme supplementation to liquid diets appears to have positively modulated the intestinal microbiota of pigs. It is interesting to note that in a similar study (conducted in the same facility and using the same diet composition where the cereals were soaked for 3 h instead of fermented) supplementation with the same exogenous enzyme appeared to negatively modulate the intestinal microbial profile^31^. Bacterial taxa that were negatively correlated with pig growth were more abundant in the ileal and caecal digesta of pigs fed the enzyme-supplemented diets (in the case of fresh diets, *Prevotellaceae* sp.,* Oscillibacter* sp. and* Rikenellaceae* sp and in the case of soaked diets, *Cellulosilyticum, Prevotellaceae* sp. and* Clostridium* sp.). This suggests that the core/basal microbial profile of the pigs used in experiments is likely to have a major impact on the results obtained and might be one of the causes of variation in results found between studies which examine the supplementation of xylanase and β-glucanase to pig diets^26,27^. Future research in this area should focus on expanding knowledge on which specific bacterial taxa in the GIT, can utilize the end-products of enzyme activity so that more precise and effective dietary supplementation strategies can be developed. With regard to cereal fermentation, there is a lack of scientific data to define optimal fermentation conditions. Therefore, further research should be performed at smaller scale to determine optimal initial fermentation time, effect of inoculant use, and to track the evolution of feed microbial profile over time.

In conclusion, cereal fermentation increased DM, OM, CP, and GE total tract nutrient digestibility and pig growth, while exogenous enzyme supplementation increased DM, OM, CP and GE ileal, and total tract digestibility resulting in improved FE. Both strategies appeared to have beneficially modulated the intestinal microbial profile in the ileum and caecum of pigs; bacterial taxa that were positively correlated with pig growth were more abundant in pigs fed the enzyme-supplemented diets, whereas most of the bacterial taxa that were negatively correlated with growth were less abundant in pigs fed the cereal-fermented diets. The results obtained help to explain the improvements found in pig growth and FE, as they indicate that beneficial bacterial taxa were more abundant in the ileum and caecum of pigs fed the enzyme-supplemented diets and that cereal fermentation reduced the abundance of intestinal bacteria that were negatively correlated with growth.

## Supplementary information

Supplementary information.

## Data Availability

The datasets used and/or analysed during the current study are available from the corresponding author on reasonable request.
