# Supplementary material for: Effect of cereal fermentation and carbohydrase supplementation on growth, nutrient digestibility and intestinal microbiota in liquid-fed grow-finishing pigs
Source: Sci Rep. 2020 Aug 13;10:13716. doi: 10.1038/s41598-020-70443-x (PMC7426827; doi:10.1038/s41598-020-70443-x)
Supplement: Supplementary file 1 — Supplementary information. [file 41598_2020_70443_MOESM1_ESM.pdf]

Supporting information for:

"Effect of cereal fermentation and carbohydrase supplementation on growth, nutrient digestibility and intestinal microbiota in liquid-fed grow-finishing pigs"

Alberto Torres-Pitarch[1,2], Gillian E. Gardiner[3], Paul Cormican[4], Mary Rea[5,6], Fiona Crispie[5,6], John V. O'Doherty[2], Pierre Cozannet[7], Tomas Ryan[1], James Cullen[3] and Peadar G. Lawlor [1,*]

[1]Teagasc, Pig Development Department, Animal and Grassland Research and Innovation Centre, Moorepark, Fermoy, County Cork, Ireland

[2]School of Agriculture and Food Science, University College Dublin, Belfield, Dublin, Ireland

[3]Department of Science, Waterford Institute of Technology, Waterford, Ireland

[4]Animal and Bioscience Research Department, Animal and Grassland Research and Innovation Centre, Teagasc, Grange, County Meath, Ireland

[5]Teagasc Food Research Centre, Moorepark, Fermoy, County Cork, Ireland

[6]APC Microbiome Ireland, University College Cork, Cork, Ireland

[7]Adisseo France SAS, Antony, France

*Correspondence: peadar.lawlor@teagasc.ie

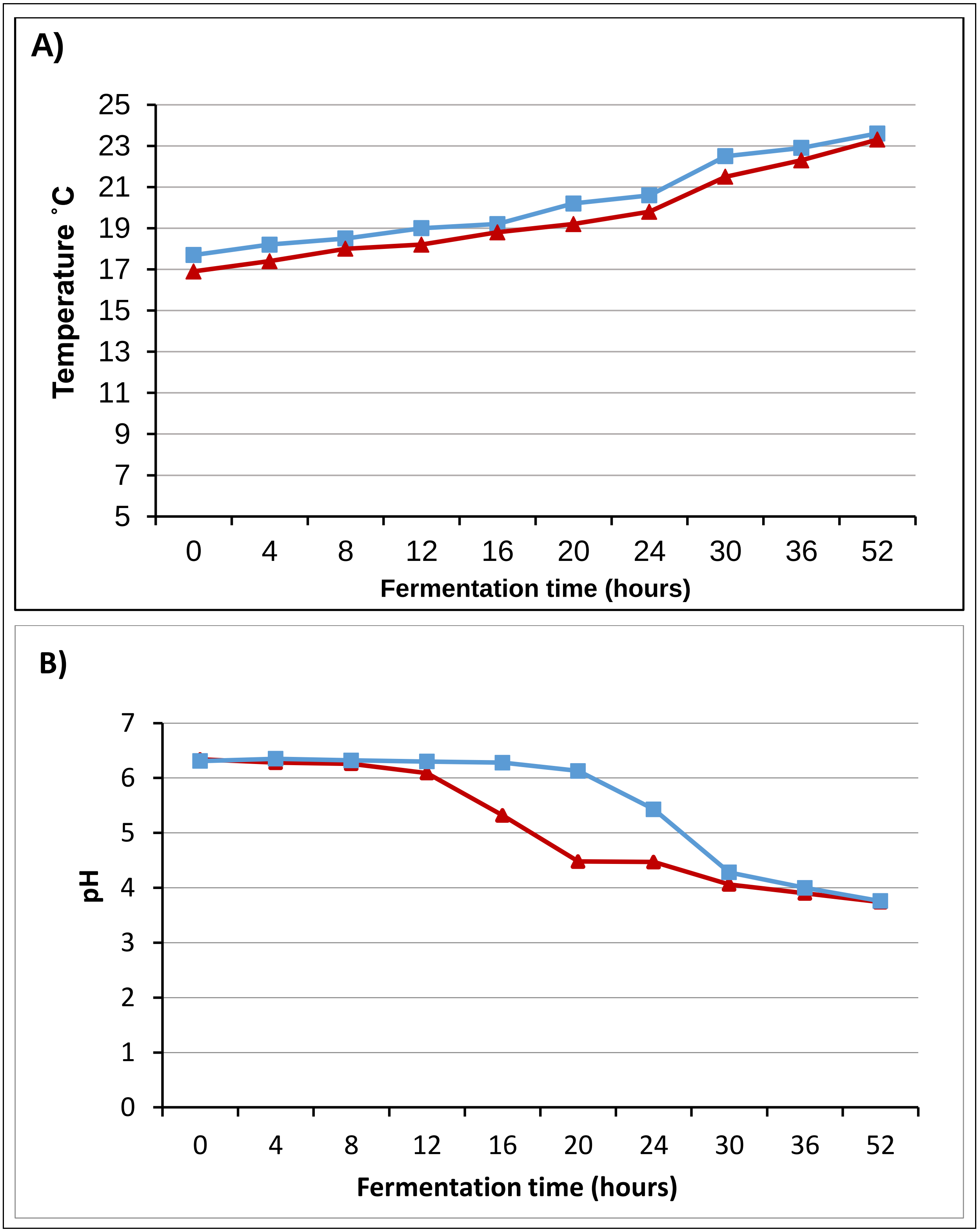
A)
Temperature ˚C
25
23
21
19
17
15
13
11
9
7
5
0 4 8 12 16 20 24 30 36 52
Fermentation time (hours)
B)
pH
7
6
5
4
3
2
1
0
0 4 8 12 16 20 24 30 36 52
Fermentation time (hours)

Figure S1. Temperature (A) and pH (B) at different time points in both fermentation tanks (▲Cereal; ■ Cereal+ENZ) during the first 52h of initial fermentation of cereals.

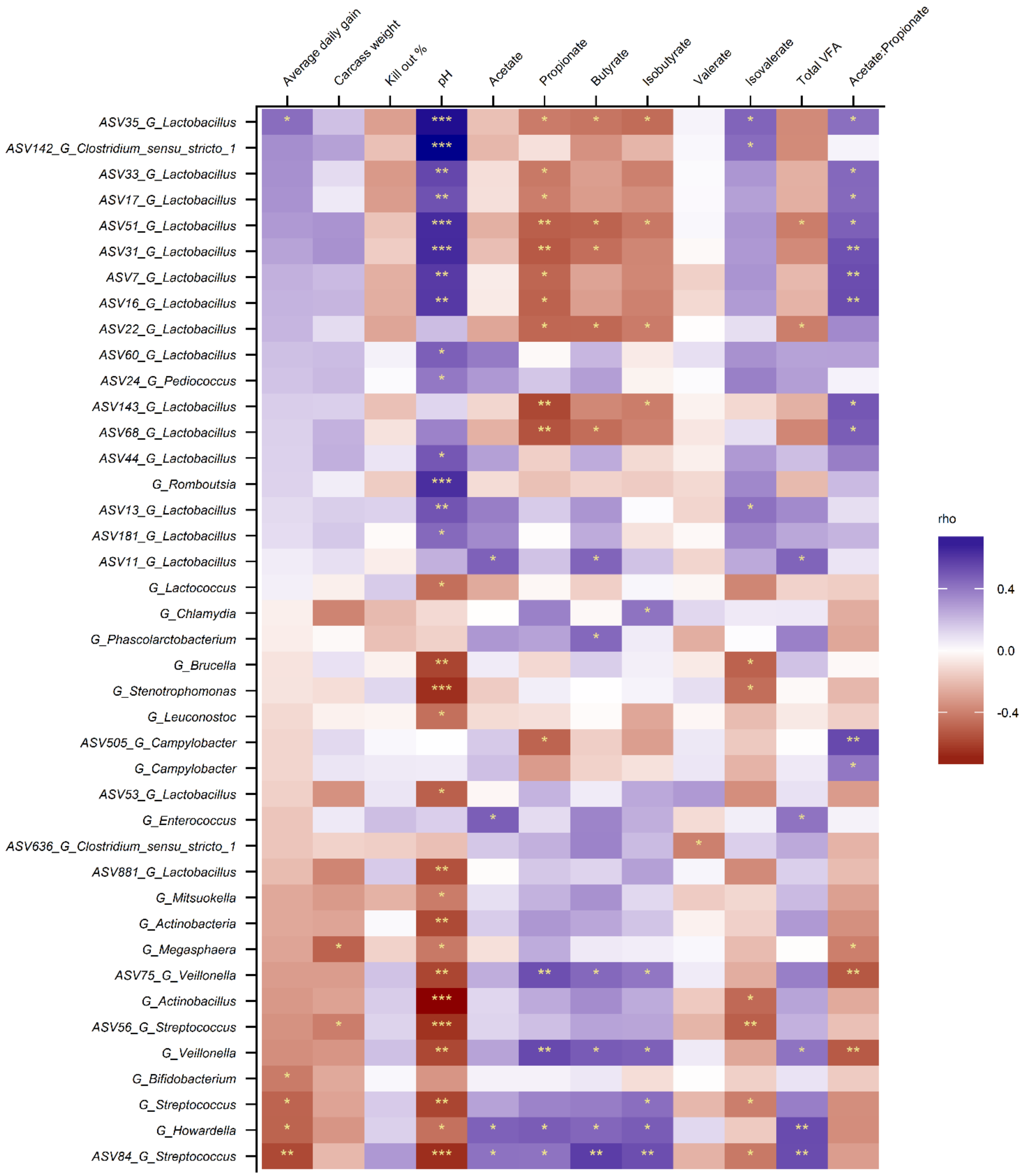

Figure S2. Spearman correlation between all ileal taxa which were differentially abundant due to treatment and all variables measured in the pigs. Positive correlations are indicated in

blue and negative correlations are indicated in red. Significant correlations are indicated with asterisks ($P<0.05$ = *, $P<0.01$ = **, $P<0.001$ = ***)

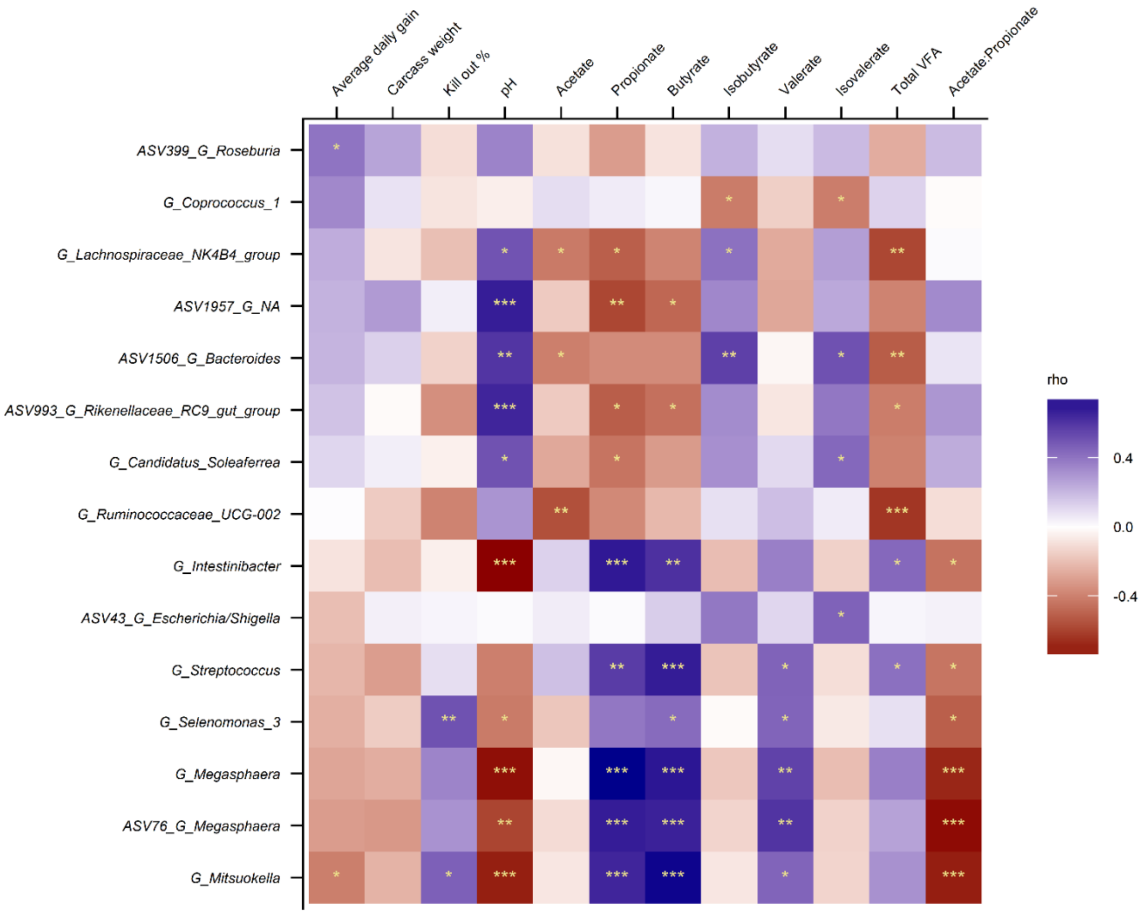

Figure S3 Spearman correlation between all caecal taxa which were differentially abundant due to treatment and all variables measured in the pigs. Positive correlations are indicated in blue and negative correlations are indicated in red. Significant correlations are indicated with asterisks (P<0.05 = *, P<0.01 = **, P<0.001 = ***)

Table S1. Effect of dietary cereal fermentation with or without carbohydrase supplementation on growth, feed intake, feed efficiency and carcass quality of grow-finisher pigs split by sex (n=9).

**Sex:**	**Females**				**Males**					**P-value**		
Cereal fermentation (**C_{ferm}**):	**Fresh**		**Ferm**		**Fresh**		**Ferm**		**SEM**	**C_{ferm}*SEX**	**ENZ*SEX**	**C_{ferm}*ENZ*SEX**
Enzyme (**ENZ**):	**-**	**+**	**-**	**+**	**-**	**+**	**-**	**+**				
Carcass weight, kg	71.9	72.3	73.9	74.0	71.7	73.4	75.1	74.1	1.00	0.870	0.899	0.332
Kill out, %	77.4	76.5	76.7	77.1	74.7	76.5	76.7	75.6	0.37	0.070	0.370	0.140
Muscle depth, mm	48.8	50.6	48.7	49.5	46.2	47.3	46.3	44.9	0.75	0.511	0.104	0.450
Fat depth, mm	10.8	11.0	11.4	11.7	11.6	12.7	12.3	12.5	0.44	0.460	0.360	0.340
Lean meat %	58.3	58.4	57.8	57.7	57.3	56.5	56.7	56.3	0.39	0.610	0.180	0.490

Table S2. Relative abundance (%) of microbial amplicon sequence variants (ASV) and families (F_) differentially abundant according to dietary treatment in the ileal and caecal digesta of pigs[1]

Cereal fermentation (C_{ferm})[2]:	**Fresh**	**Fresh**	**Ferm**	**Ferm**		**P-value[5]**		
Enzyme (ENZ)[3]:	**-**	**+**	**-**	**+**	**SEM[4]**	**ENZ**	**C_{ferm}**	**ENZ*C_{ferm}**
**Ileum**								
F_Enterococcaceae	1.8[a]	0.93[a]	0.05[a,b]	0.01[b]	2.500	0.97	<0.05	0.10
F_Leuconostocaceae	0.25[a,b]	0.29[a]	0.1[b]	0.10[b]	0.089	0.91	<0.05	0.10
F_Acidaminococcaceae	0.07[a]	0.01[a,b]	0.01[a,b]	0.00[b]	0.033	0.28	<0.05	0.10
F_Xanthomonadaceae	0.13[a]	0.1[a]	0.03[a,b]	0.01[b]	0.060	0.61	<0.05	0.07
F_Prevotellaceae	0.13[a,b]	0.19[a]	0.02[c]	0.05[cb]	0.081	0.70	<0.05	<0.05
F_Mycoplasmataceae	0.01[b]	0[b]	0.19[a]	0.04[a,b]	0.098	0.45	<0.05	<0.05
F_Chlamydiaceae	0.1[a]	0.01[a,b]	0.05[a]	0.00[b]	0.064	0.28	0.50	<0.05
F_Pasteurellaceae	0.78[a]	1.01[a]	0.09[a,b]	0.02[b]	0.627	0.98	<0.01	<0.05
F_Brucellaceae	0.02[a]	0.02[a]	0.01[b]	0.00[b]	0.008	0.76	<0.01	<0.05
F_Campylobacteraceae	0.02[c]	0.03[cb]	0.07[a,b]	0.20[a]	0.086	0.37	<0.01	0.07
F_Bifidobacteriaceae	0.11[a]	0.31[a]	0.01[b]	0.01[b]	0.146	0.37	<0.01	<0.01
F_Veillonellaceae	2.45[a]	1.1[a]	0.63[a]	0.09[b]	1.158	0.35	<0.05	<0.05
F_Streptococcaceae	7.43[a]	2.19[a]	0.28[b]	0.08[b]	3.411	0.35	<0.01	<0.01
ASV_107	0.09[a,b]	0.03[b]	0.01[c]	0.32[a]	0.102	0.38	0.72	<0.05
ASV_11	0.13[bc]	0.18[b]	0.48[a]	0.09[c]	0.102	0.08	0.15	<0.01
ASV_12	0.86[b]	1.91[b]	11.82[a]	0.76[b]	4.460	<0.05	0.05	<0.01
ASV_13	0.21[b]	0.34[b]	2.78[a]	0.06[c]	1.017	<0.01	0.08	<0.01
ASV_142	0.09[b]	0.11[b]	0.27[a]	0.25[a]	0.072	0.96	<0.05	0.06
ASV_143	0.05[c]	0.18[b]	0.04[c]	0.36[a]	0.049	<0.01	0.09	<0.01
ASV_159	0.03[a]	0.04[a]	0.01[b]	0.01[b]	0.012	0.80	<0.01	<0.05
ASV_16	0.28[b]	0.7[b]	0.72[b]	2.63[a]	0.940	0.09	0.06	<0.05
ASV_17	0.63[b]	1.14[b]	0.42[b]	6.25[a]	2.707	<0.01	0.09	<0.05
ASV_18	0.04[b]	0.03[b]	0.2[a]	0.04[b]	0.050	<0.05	<0.05	<0.05
ASV_181	0.03[b]	0.06[b]	0.26[a]	0.00[c]	0.049	0.13	0.12	<0.05
ASV_188	0.01[b]	0.01[b]	0.13[a]	0.04[a,b]	0.044	0.48	<0.05	0.05
ASV_22	0.37[b]	1.04[b]	0.06[c]	4.67[a]	1.752	<0.01	0.16	<0.01
ASV_226	0.02[b]	0.01[b]	0.02[b]	0.32[a]	0.112	<0.05	<0.01	<0.01
ASV_24	0.40[b]	0.87[b]	5.42[a]	0.35[b]	2.059	<0.05	0.05	<0.01
ASV_25	0.11[b]	0.16[b]	1.36[a]	0.03[c]	0.495	<0.05	0.06	<0.01
ASV_259	0.00[c]	0.00 [c]	0.01[a]	0.01[b]	0.000	0.45	0.15	0.05
ASV_31	0.15[c]	0.36[b]	0.4[b]	2.16[a]	0.589	<0.01	<0.01	<0.01
ASV_33	0.45[b]	0.59[b]	0.23[b]	2.83[a]	0.941	<0.05	0.12	<0.05
ASV_35	0.18[b]	0.86[a]	1.36[a]	2.17[a]	0.828	0.42	0.06	<0.05
ASV_36	0.17[c]	0.59[b]	3.08[a]	0.08[c]	1.074	<0.05	0.10	<0.01
ASV_44	0.09[b]	0.15[b]	0.96[a]	0.01[c]	0.204	<0.05	0.22	<0.01
ASV_505	0.01[c]	0.03[cb]	0.1[a,b]	0.18[a]	0.080	0.62	<0.05	<0.05
ASV_51	0.09[c]	0.20[b]	0.24[b]	0.94[a]	0.186	<0.05	<0.01	<0.01
ASV_53	1.57[a]	0.60[a]	0.56[a]	0.07[b]	0.686	0.21	0.11	<0.05
ASV_56	0.91[a]	1.16[a]	0.11[b]	0.03[b]	0.734	0.86	<0.05	0.05

Cereal fermentation (C_{ferm})[2]:	**Fresh**	**Fresh**	**Ferm**	**Ferm**		**P-value[5]**		
Enzyme (ENZ)[3]:	**-**	**+**	**-**	**+**	**SEM[4]**	**ENZ**	**C_{ferm}**	**ENZ*C_{ferm}**
ASV_60	0.04[bc]	0.10[b]	0.42[a]	0.02[c]	0.097	0.08	0.17	<0.05
ASV_636	0.09[a]	0.08[a]	0.01[b]	0.01[b]	0.033	0.96	<0.05	0.07
ASV_68	0.08[c]	0.22[b]	0.09[bc]	0.8[a]	0.242	<0.01	0.08	<0.01
ASV_7	0.49[c]	1.53[b]	1.49[b]	5.93[a]	2.124	<0.05	0.05	<0.05
ASV_75	1.09[a]	0.47[a]	0.52[a]	0.07[b]	0.501	0.29	0.15	<0.05
ASV_83	0.1[b]	1.9[a]	0.08[b]	0.43[a,b]	0.943	<0.05	0.16	<0.05
ASV_84	0.58[a]	0.76[a]	0.1[b]	0.02[b]	0.314	0.83	<0.05	<0.05
ASV_881	0.07[a]	0.04[a]	0.06[a]	0.01[b]	0.011	0.31	0.58	<0.05
**Caecum**								
F_Defluviitaleaceae	0.05[a]	0[c]	0.01[b]	0.01[b]	0.008	0.19	0.34	<0.05
F_Veillonellaceae	7.68[a]	0.21[c]	4.99[a]	2.72[b]	1.223	0.19	0.12	<0.05
F_Streptococcaceae	0.73[a]	0.94[a]	0.11[b]	0.01[c]	0.467	0.91	0.01	<0.01
F_Enterobacteriaceae	0.32[b]	1.55[a]	0.07[c]	0.1[c]	0.603	0.19	<0.01	<0.01
ASV_1049	0.01[c]	0.09[a]	0.05[b]	0.05[b]	0.011	0.54	0.79	<0.01
ASV_12	0.13[bc]	0.3[b]	0.96[a]	0.07[c]	0.347	0.45	0.53	<0.01
ASV_1506	0.02[b]	0.04[a]	0[c]	0.04[a]	0.010	0.30	0.92	<0.01
ASV_1562	0.03[a]	0.03[a]	0.01[b]	0.01[b]	0.006	0.62	<0.01	0.16
ASV_178	0.58[a]	0.05[b]	0.1[b]	0.1[b]	0.165	<0.01	0.29	<0.01
ASV_1957	0[c]	0[cb]	0.02[a,b]	0.03[a]	0.011	0.49	<0.01	0.13
ASV_203	0.07[b]	0.28[a]	0.08[b]	0.34[a]	0.112	<0.01	0.92	0.14
ASV_26	0.19[b]	0.92[a]	0.1[bc]	0.06[c]	0.312	0.53	<0.01	<0.01
ASV_2740	0.01 [d]	0.02[c]	0.04[a]	0.03[b]	0.000	0.93	0.44	<0.01
ASV_36	0.04[b]	0.1[b]	0.23[a]	0.01[c]	0.000	0.49	0.61	<0.01
ASV_399	0.04[b]	0.19[a]	0.04[b]	0.18[a]	0.000	<0.01	0.83	0.16
ASV_43	0.13[b]	0.56[a]	0.04[c]	0.03[c]	0.000	0.47	<0.01	<0.01
ASV_76	0.97[a]	0.47[a]	0.39[a]	0.01[b]	0.000	0.60	0.50	<0.01
ASV_993	0.25[a]	0.02[c]	0.08[b]	0.03[c]	0.000	<0.01	0.64	<0.01

[1] Values within a row that do not share a common superscript are statistically different (P < 0.05).
[2] Fermenting the cereal fraction of the diet prior to feeding (fresh = un-fermented, ferm = fermented).
[3] Enzyme supplementation with a xylanase and β-glucanase complex [unsupplemented (-), supplemented (+)].
[4] SEM = standard error of the mean.
[5] P-value corrected for false discovery rate (FDR).
